# Mapping Temperature Heterogeneities during Catalytic CO_2_ Methanation with *Operando* Luminescence Thermometry

**DOI:** 10.1021/acsnano.3c05622

**Published:** 2023-10-05

**Authors:** Thimo S. Jacobs, Thomas P. van Swieten, Sander J. W. Vonk, Isa P. Bosman, Angela E. M. Melcherts, Bas C. Janssen, Joris C. L. Janssens, Matteo Monai, Andries Meijerink, Freddy T. Rabouw, Ward van der Stam, Bert M. Weckhuysen

**Affiliations:** † Inorganic Chemistry and Catalysis, Debye Institute for Nanomaterials Science & Institute for Sustainable and Circular Chemistry, Utrecht University, Universiteitsweg 99, 3584 CG Utrecht, The Netherlands; ‡ Condensed Matter and Interfaces, Debye Institute for Nanomaterials Science, Utrecht University, Princetonplein 1, 3584 CC Utrecht, The Netherlands; § Soft Condensed Matter and Biophysics, Debye Institute for Nanomaterials Science, Utrecht University, Princetonplein 1, 3584 CC Utrecht, The Netherlands

**Keywords:** luminescence thermometry, CO_2_ methanation, heterogeneous catalysis, temperature uncertainty, lanthanides

## Abstract

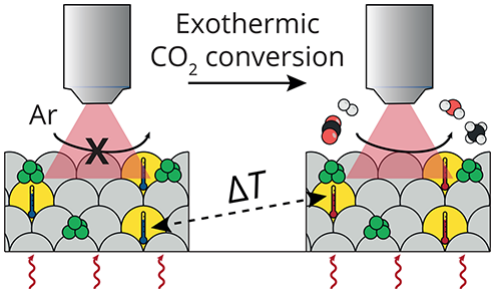

Controlling and understanding reaction temperature variations in catalytic processes are crucial for assessing the performance of a catalyst material. Local temperature measurements are challenging, however. Luminescence thermometry is a promising remote-sensing tool, but it is cross-sensitive to the optical properties of a sample and other external parameters. In this work, we measure spatial variations in the local temperature on the micrometer length scale during carbon dioxide (CO_2_) methanation over a TiO_2_-supported Ni catalyst and link them to variations in catalytic performance. We extract local temperatures from the temperature-dependent emission of Y_2_O_3_:Nd^3+^ particles, which are mixed with the CO_2_ methanation catalyst. Scanning, where a near-infrared laser locally excites the emitting Nd^3+^ ions, produces a temperature map with a micrometer pixel size. We first designed the Y_2_O_3_:Nd^3+^ particles for optimal temperature precision and characterized cross-sensitivity of the measured signal to parameters other than temperature, such as light absorption by the blackened sample due to coke deposition at elevated temperatures. Introducing reaction gases causes a local temperature increase of the catalyst of on average 6–25 K, increasing with the reactor set temperature in the range of 550–640 K. Pixel-to-pixel variations in the temperature increase show a standard deviation of up to 1.5 K, which are attributed to local variations in the catalytic reaction rate. Mapping and understanding such temperature variations are crucial for the optimization of overall catalyst performance on the nano- and macroscopic scale.

## Introduction

Solid catalysts are typically heterogeneous in composition, size, and shape.^1−3^ They consist of components ranging from the nano- to millimeter size range, so variations in the density of active sites could be present on the sub-micrometer scale.^4^ It is expected that the heterogeneity of the catalyst leads to spatial variations in reaction rates of endo- or exothermic reactions and therefore temperature variations and hot spots.^5^ These variations can, as shown in the Arrhenius equation, impact the activity and selectivity of the catalyst on a macroscopic scale. However, directly detecting these spatial variations on a micrometer scale is difficult.^6,7^

Luminescence thermometry is a technique that promises to image local temperature variations on the micrometer scale. This technique relies on the temperature-dependent emission from nanoscale probes to achieve thermal sensing.^8^ Luminescence thermometry experiments during a catalytic reaction are challenging: they require the light-emitting material to operate under difficult conditions.^9,10^ In particular, catalytic reactions are often performed at elevated temperatures of up to several hundred degrees Celsius, where many luminescent materials are quenched.^11,12^ Lanthanide-doped materials are ideally suited for thermometry at such elevated temperatures, because their emission can remain relatively bright by tuning the composition of the material.^13^ However, the precision of such temperature measurements depends on various considerations, such as the lanthanide ion used, the host material, and the doping concentration.^14^ The optimal thermometer material further depends on the temperature regime of interest. Selecting the best material for studying temperature fluctuations in catalytic materials at elevated temperatures is therefore not trivial.^15,16^

While previous work used a spot size of a millimeter to measure the temperature during catalytic reactions, temperature variations on a smaller size scale are also highly relevant for catalytic reactions.^7,17^ Measuring at high spatial resolutions requires the collection of sufficient luminescence signal from small volumes of sample,^18,19^ so the selection of bright thermometer material is important. The desire for a high spatial resolution may further lead to prolonged measurement durations in a scanning procedure; therefore, the mechanical stability of the setup becomes critical, especially under catalytic working conditions. Finally, the inhomogeneity of the catalyst on the microscopic scale can lead to spatially varying absorption and/or scattering effects, which potentially affects temperature readout.^20^ These challenges have until now hindered the high-spatial-resolution *operando* luminescence thermometry of catalysts.

In this work, we measure spatial variations in temperature on the micrometer scale during the catalytic CO_2_ hydrogenation reaction using neodymium-based luminescence thermometry and link them to variations in catalytic activity. This reaction is typically performed between 473 and 673 K and is relevant for H_2_ purification in ammonia synthesis or for the storage of solar and wind energy in power-to-gas schemes.^21−24^ Here, we mixed a Ni/TiO_2_ catalyst with luminescent Nd^3+^-doped Y_2_O_3_. Using a small reaction cell loaded on a commercially available microscope for Raman spectroscopy and imaging, we map local temperatures during the CO_2_ methanation reaction at set temperatures of 573, 623, and 673 K.^25^ We first carefully minimize and quantify temperature uncertainties due to measurement noise as well as systematic errors due to scattering, absorption, drift, and background fluorescence by the catalyst sample. The average increase in temperature over all pixels amounts to 6–25 K, increasing with the average reactor set temperature. We can then identify the spatial variations in temperature on length scales of a micrometer, amounting to up to 1.5 K (standard deviation). These are likely the result of spatial variations in catalytic activity, for example, caused by variations in catalyst density. Using these results, luminescence thermometry could help in understanding the catalyst at work by, for instance, considering local variations in temperature on the overall reaction rate and selectivity.

## Results and Discussion

### Boltzmann Thermometry over a Broad Temperature Range

**Figure 1:**
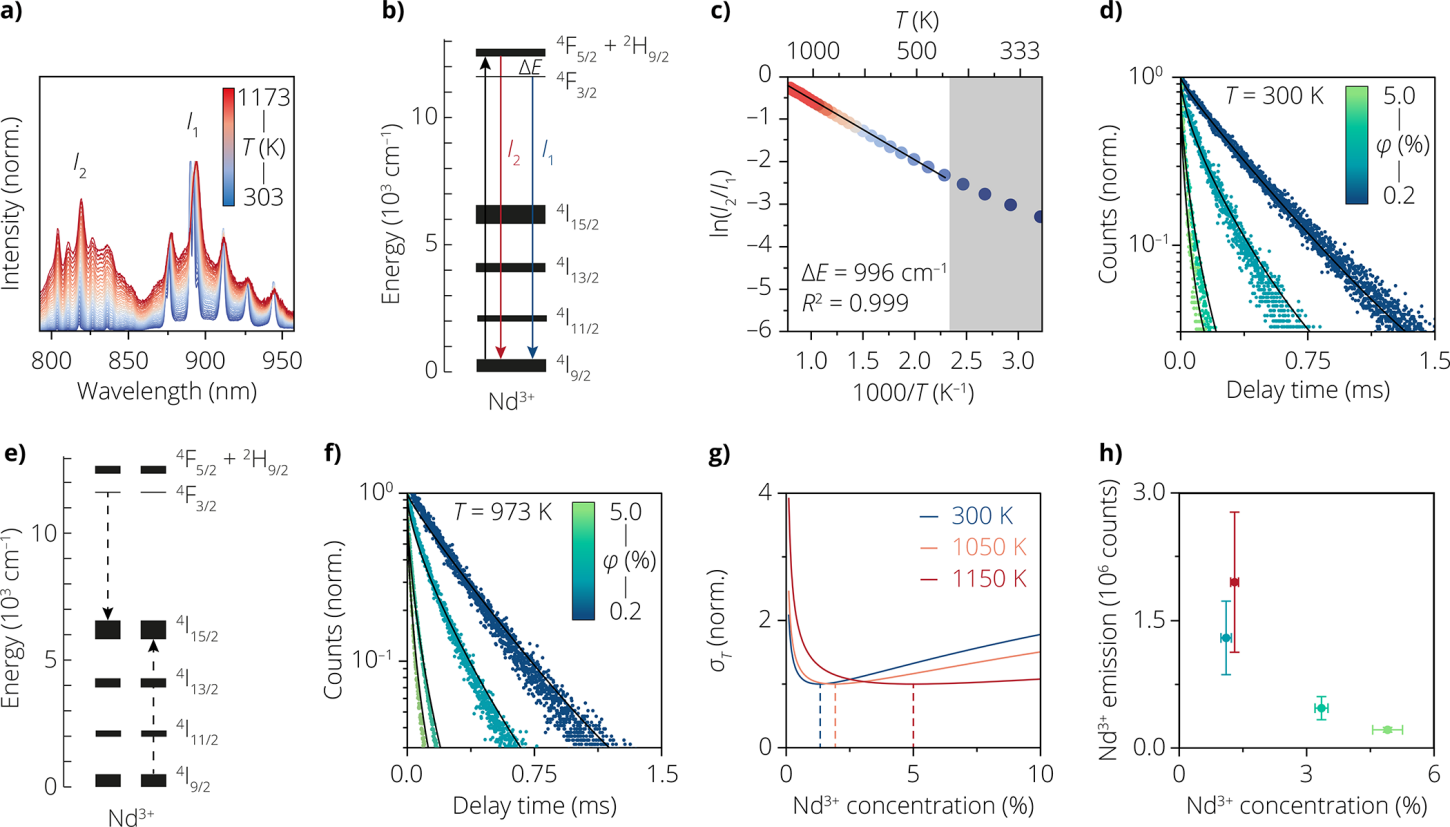
(a) Spectra of the temperature-dependent emission from microcrystalline Y_2_O_3_ doped with 3.4% of Nd^3+^, collected for 785 nm excitation. Background blackbody spectra were collected and subtracted above 963 K (see Figure S2). (b) Energy-level diagram of a single Nd^3+^ ion, with the black arrow representing the 785 nm excitation and the colored arrows marking the radiative emission of *I*_2_ (red) and *I*_1_ (blue) as also shown in (a), respectively. (c) Natural logarithm of the integrated intensity ratio between *I*_2_ (795–860 nm) and *I*_1_ (860–960 nm) versus the inverse temperature 1/*T* for the spectra in (a) and fitted to the Boltzmann model (black line). (d) Photoluminescence decay curves of the ^4^F_5/2_ energy level at different concentrations of Nd^3+^ (0.2%, 1.1%, 3.4%, and 4.9% going from blue to green, respectively; see Table S1) at room temperature. The solid black lines are the result of a global fit of all of the decay curves to a cross-relaxation model. (e) Energy diagram of two Nd^3+^ ions. The dashed arrows represent cross-relaxation from the ^4^F_3/2_ level to the ^4^I_15/2_ level of an excited Nd^3+^ ion, promoting a nearby ground-state Nd^3+^ ion to the ^4^I_15/2_ level. (f) Same as in (d), but measured at 973 K. The solid lines are calculated decay curves using the cross-relaxation strength determined in (d). (g) Calculated values for the temperature uncertainties in different temperature regimes, 300 K (blue), 1050 K (orange), and 1150 K (red), respectively. The temperature uncertainties are normalized to the minimum value at every temperature. These relative temperature uncertainties allow us to compare dopant concentrations, independent of various experimental parameters, such as measurement duration. Dashed lines indicate the optimal neodymium concentration for that temperature regime. (h) Neodymium emission intensity between 795 and 960 nm at room temperature, measured on the same microscope as used for the experiments below. The vertical error is the standard deviation in emission intensity over a 70 × 70 μm area. The horizontal error bar is the uncertainty in Nd^3+^ concentration from the ICP-OES measurements in Table S1.

For luminescence thermometry, we use temperature-dependent luminescence from Nd^3+^-doped Y_2_O_3_ particles with a diameter of around 1 μm (see Figure S1 for structural characterization). Figure 1a shows the temperature-dependent emission spectra of these luminescent thermometers excited at 785 nm. We observe emissions from two thermally coupled excited states to the ground state: from the ^4^F_3/2_ level at 860–960 nm and from the ^4^F_5/2_ level at 795–860 nm. Figure 1b shows the energy-level scheme of Nd^3+^ and the relevant absorption and emission transitions. With increasing temperature, the emission intensity of the spectral area *I*_2_ increases compared to *I*_1_, reflecting a change in relative populations of the two emitting levels. More specifically, over the temperature range of 400–1173 K, the natural logarithm of the luminescence intensity ratio, ln(LIR) = ln(*I*_2_/*I*_1_), scales linearly with inverse temperature (Figure 1c). This evidences that the excited-state populations follow Boltzmann statistics. Deviations from the linear trend at lower temperatures (*T* < 400 K) are due to slow coupling between the emitting levels compared to the depopulation rate.^26−28^ As our catalytic reactions take place at high temperatures of *T* > 400 K, we have to consider only the high-temperature properties of the thermometer.^29,30^ We calibrate the thermometer material by fitting the linear part of the data (*T* ≥ 423 K) in Figure 1c to a Boltzmann model (Figure S2)
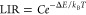
1where *k*_B_ is Boltzmann’s constant and *C* is a prefactor that depends on the radiative decay rates and the degeneracy of the emitting levels, transmission of the sample, and detector efficiency. The fitted energy difference between the emitting levels of Δ*E* = 996 cm^–1^ is consistent with the literature.^31^ This value for Δ*E* will be used in the remainder of our work to convert the LIR to temperature maps.

### Quantifying the Ion–Ion Interactions

For optimal signal strengths during *operando* thermometry experiments, we want to find the Nd^3+^ doping concentration that produces the brightest emission. Two counteracting effects determine the optimum doping concentration.^32^ On the one hand, higher concentrations lead to brighter emission simply because there are more emitting Nd^3+^ ions. On the other hand, if Nd^3+^ ions are packed more closely together in the host crystal, ion–ion interactions lead to emission quenching.^33^

Figure 1d shows the photoluminescence decay curves at room temperature of Y_2_O_3_ doped with an increasing Nd^3+^ doping concentration. Faster and more multiexponential decay is observed for the higher concentration Nd^3+^ samples. This is consistent with cross-relaxation, where a Nd^3+^ ion in one of the emitting states can transfer part of its energy to a neighboring Nd^3+^ ion in the ground state (Figure 1e). This quenches the emission and shortens the lifetime of the emitting state. Using a model with discrete ion-to-ion distances and a global-fitting procedure, we extract the cross-relaxation strength (eq S2). The strength of cross-relaxation is independent of the temperature, as shown in Figure 1f. Using these parameters, we can then calculate the emission efficiency as a function of doping concentration (eqs S4–S6). Since the catalytic reaction of interest in this work is performed below 900 K, thermal quenching does not yet have a negative effect on the emission signal (Figure S4a).

### Minimizing Temperature Uncertainty

Our model of emission efficiency as a function of the doping concentration allows us to predict the dopant concentration that produces the brightest signal. More specifically, we consider the estimated temperature uncertainty (σ_*T*_) as the relevant figure of merit, which is inversely proportional to the square root of the emission efficiency and to the square root of number of emitting ions (eq S7). Figure 1g shows the expected temperature uncertainties as a function of the doping concentration. Between 300 and 900 K, the emission efficiency is determined by a competition between the radiative decay and cross-relaxation. The optimal concentration of neodymium is 1.3%. If we wanted to perform catalytic reactions at higher temperatures, the optimal concentration of neodymium would shift to higher Nd^3+^ concentrations, caused by a change in the balance between the factors mentioned above because of additional thermal quenching. Figure 1f also illustrates this thermal quenching by a shortening of the lifetime of the excited states. The change becomes even more apparent at 1150 K, where 5.0% neodymium results in the lowest temperature uncertainty. For our CO_2_ methanation experiments between 573 and 673 K, we synthesized a thermometer with a neodymium concentration of 1.2% and this sample will be used to study the local temperature during the catalytic reaction (Figure 1h).

### Measuring Spatial Temperature Variations during Catalysis

We can now apply the optimized luminescence thermometer, Y_2_O_3_:Nd^3+^ (1.2%, Table S1), for *operando* temperature measurements during catalytic CO_2_ hydrogenation experiments. The desired CO_2_ hydrogenation reaction toward methane is an exothermic reaction given by
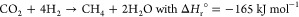
2

**Figure 2:**
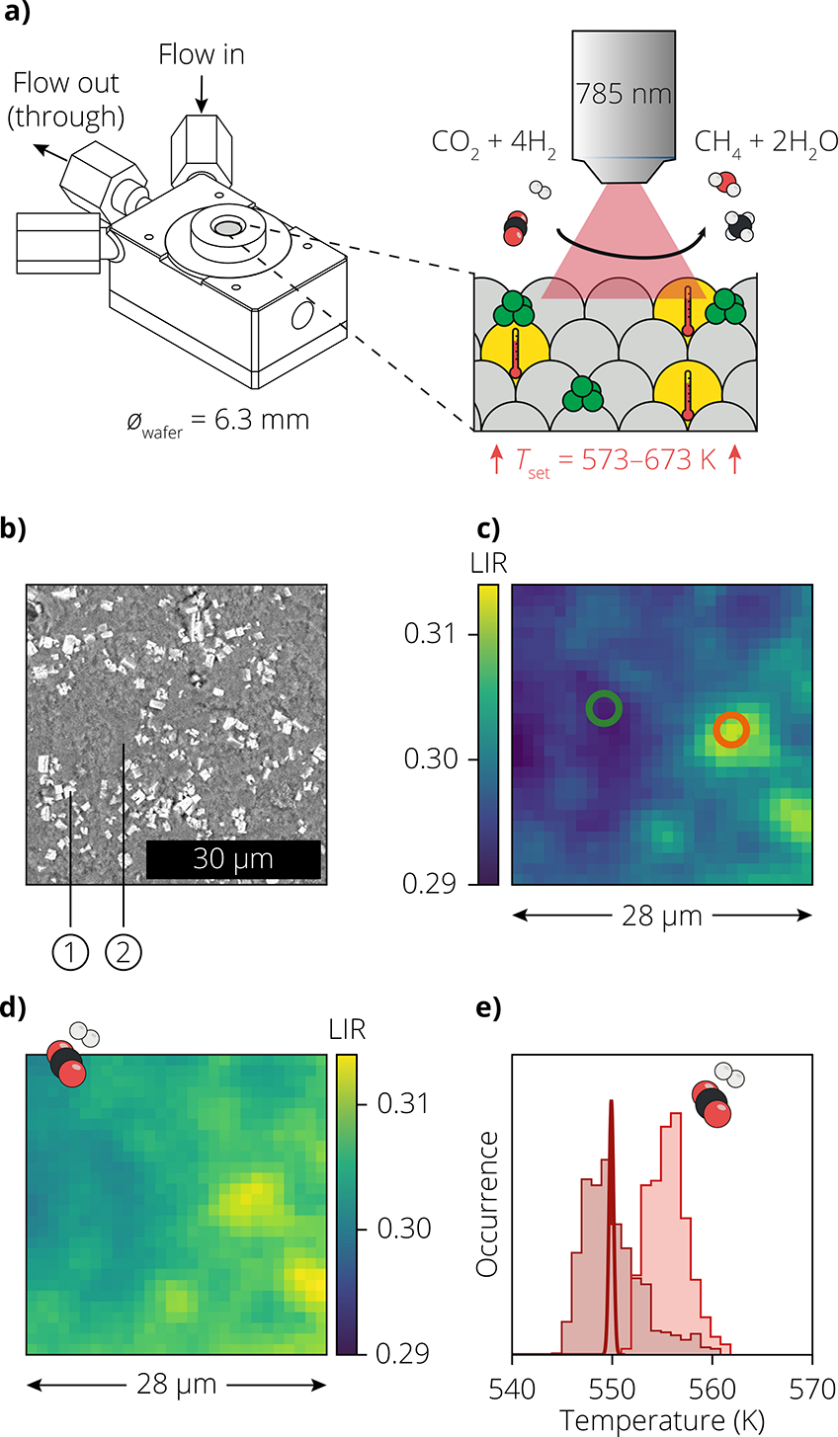
(a) Measurement cell loaded with a catalyst containing 6 wt % Ni supported on TiO_2_, mixed in 4:1 ratio with Y_2_O_3_:Nd^3+^ (1.2 wt %) thermometer particles. (b) Scanning electron microscope (SEM) image of the sample, with thermometry particles (1) and catalyst material (2). (c) Two-dimensional (2D) map of the measured luminescence intensity ratios (LIR) under an Ar atmosphere on a 28 × 28 μm area at a reactor temperature of 550 K. The integration time per pixel was 0.05 s. (d) Same as in (c), but after the addition of CO_2_ and H_2_ to the gas feed. (e) Histogram of the calculated temperatures from the LIR values in (c) (dark red) and (d) (light red), analyzed using a single value for the prefactor *C* of eq 1. The Gaussian centered at 550 K is the distribution of temperature values expected for the measurement in Ar, based on the reactor temperature and the estimated temperature uncertainty.

The catalyst of choice to perform the CO_2_ hydrogenation is a TiO_2_ support with 6 wt % of Ni (characterization in Figure S1). The catalyst was mixed with Y_2_O_3_ thermometry particles by grinding (1:4 weight ratio, thermometers to catalyst) and pressed into a self-supporting wafer where the thermometry particles were in the near vicinity of the catalyst particles to ensure optimal heat transfer and hence local temperature readout. Y_2_O_3_ has been reported to have no catalytic activity under the conditions used.^22^ The sample was loaded into a commercial Harrick measurement cell and reduced under an H_2_ and Ar atmosphere (Figure 2a,b). After reduction, the set temperature in the measurement cell was increased (to 573, 623, or 673 K) while keeping a constant presence of Ar. We calibrated that the actual temperatures at the location of the sample, which we will call the “reactor temperatures” in the remainder of this work, reach slightly lower than the set temperatures (Figure S15). A 785 nm excitation laser, which is conveniently available on our commercial microscope used for Raman microscopy, was focused and scanned over the surface with a step size of 1.0 μm, obtaining the emission spectra of neodymium. Next, CO_2_ and H_2_ were introduced to initiate the reaction and the scan was repeated on the same area.

Figure 2c shows the map of the LIR under an inert atmosphere of Ar at a reactor temperature of 550 K. The pixel size is 1.0 × 1.0 μm over a field of view of 28 × 28 μm. We observe that there are spatial variations in the LIR. As we introduce the reaction gas mixture of H_2_ and CO_2_, the average LIR of this region rises (Figure 2d), indicating increasing temperatures due to heat generation by the catalytic reaction. Mass spectrometry confirmed that the introduction of reaction gases results in the production of CH_4_ (Figure S6a). We convert the LIR values to apparent temperatures using eq 1 with the same value of the prefactor *C* for every pixel. A histogram of the apparent temperatures over the 28 × 28 μm area shows temperature variations much wider than expected based on photon-counting noise (Figure 2e; see also Figure S7). While the temperature variations during the reaction (Figure 2d) could be due to heterogeneities in catalyst activity, variations under an inert atmosphere (Figure 2c) are not expected.

As random photon-counting noise or reaction-induced heating cannot explain the variations in apparent temperature under inert conditions, we propose different possible origins: spatial variations in (1) absorption, (2) scattering of thermometer emission by the sample, or (3) background fluorescence. Local variations in absorption and/or scattering at the wavelengths monitored to determine *I*_1_ and *I*_2_ would lead to deviations of the recorded LIR from the emitted LIR.^34^ Moreover, the fluorescence background from the sample could add to *I*_1_ and *I*_2_ and therefore affect the LIR. These variations result in artifacts in the temperature mapping experiments, which do not reflect actual local temperature variations, but they appear as such. Local variations in absorption, scattering, and background fluorescence therefore require careful characterization while determining spatial temperature variations. A similar issue was previously identified in biological experiments, where wavelength-dependent transmission of tissue was identified as a complicating issue.^20^ Indeed, the Ni/TiO_2_ catalyst turns from green to black upon reduction (Figure S8), making local variations in the light absorption likely. The contribution of scattering is evidenced by reference measurements on a colorless thermometer sample without catalyst, showing variations in the LIR of the Nd^3+^ emission (Figure S9). Moreover, reference measurements on a sample with undoped Y_2_O_3_ material and reduced catalyst shows spatial variations of background fluorescence (Figure S10). The variations in LIR are also observed at room temperature without a gas flow (Figure S11).

**Figure 3:**
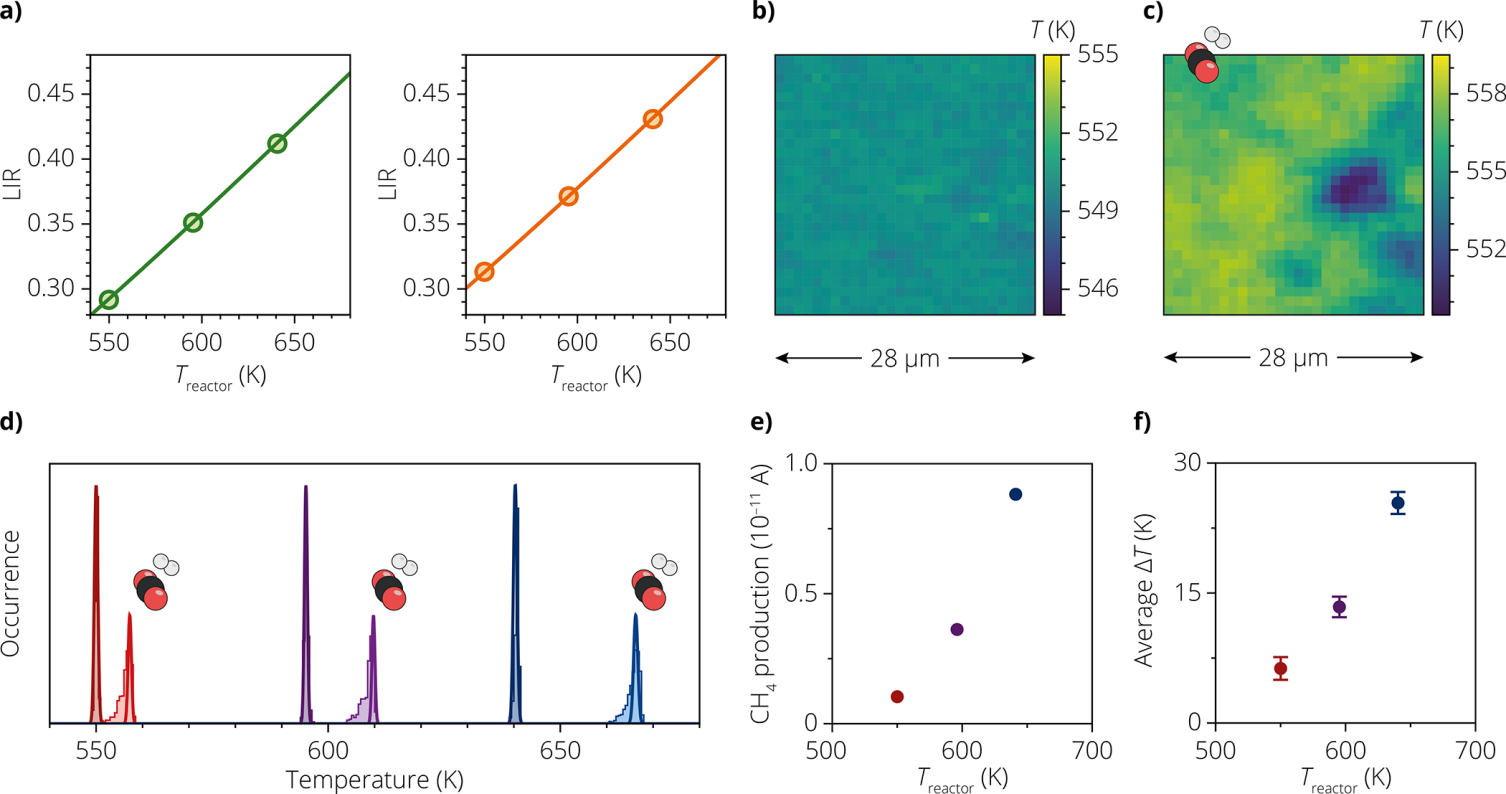
(a) Local calibrations at the two pixels marked in Figure 2c, using the fit model of eq 3 and LIR values corrected for small sample drift between measurements (see page 16 in the Supporting Information). (b) 2D map of the temperatures measured in only Ar at a reactor temperature of 550 K, calculated from the LIR values in Figure 2c and using the local drift-corrected calibration. (c) Same as in (b), but after the introduction of CO_2_ and H_2_. (d) Histograms of the local temperatures recorded at different reactor temperatures (550, 595, and 640 K, going from red to blue). The histograms indicated with the CO_2_ and H_2_ molecules are measured during the catalytic reaction, while the histograms without molecules are measured under inert conditions. The Gaussian solid lines show the expected width of the histogram due to measurement uncertainties. The Gaussian solid lines of the measurements with the reaction gas are shifted to match the maximum of the extracted readout temperature distribution. (e) CH_4_ production from mass spectrometry (*m*/*z* = 15) at different reactor temperatures. (f) Average local temperature increase at the different reactor temperatures under reaction conditions, obtained using the local-calibration procedure (eq 3). The error bars denote the magnitudes of spatial variations (one standard deviation). These are the variations, σ_T,corrected_, which are corrected for the measurement uncertainty.

To correct for local variations in absorption, scattering, and fluorescence, we performed a local calibration. Specifically, for each pixel in the map, we recorded the LIR during three reference measurements under an inert atmosphere at known reactor temperatures of 550, 595, and 640 K. We fit these three reference LIR values to the equation
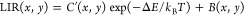
3where the spatially dependent prefactor *C′*(*x*,*y*) differs from *C* due to local absorption and scattering of emitted light and the term *B*(*x,y*) describes the local contribution of background fluorescence to the LIR. See pages 12–14 in the Supporting Information for further discussion of eq 3. Figure 3a shows two examples of local calibration curves, recorded at the pixels marked in Figure 2c.

Figure 3b,c shows temperature maps constructed based on the local calibrations of spectral distortions and fluorescence background. The maps were recorded at a reactor temperature of 550 K and under an inert atmosphere (Figure 3b) or under a flow of reaction gases (Figure 3c). See Figure S12 for the maps recorded at other reactor temperatures and for a description of the order of the experiment series. The map recorded under an inert atmosphere (Figure 3b) shows a homogeneous temperature equal to the reactor temperature, as expected, because no catalytic reaction takes place. The spatial variations in the temperature map amount to a standard deviation of σ_*T*_ = 0.31 K, which matches the uncertainty of δ_*T*_ = 0.37 K estimated by using error propagation (page 17 in the Supporting Information). When reaction gases are introduced, the temperatures are higher by an average of 6.3 K (Figure 3c), which is expected because the reaction is exothermic.^35,36^ Spatial variations are obvious in the temperature map during reaction. The spatial variations in the temperature readout are clearly not random. Indeed, the standard deviation in temperature of σ_*T*_ = 1.4 K is significantly larger than the estimated uncertainty of δ_*T*_ = 0.36 K. We therefore conclude that the map shows real spatial temperature variations caused by spatial variations of the catalytic activity. After correcting for random noise due to measurement uncertainty, the magnitude of real temperature variations is still estimated at . The temperature controller of the Harrick cell did not record a temperature increase upon introduction of reaction gases. This highlights how luminescence thermometry provides otherwise inaccessible information about reaction-induced heating. It also implies that our studies of local heating are unaffected by the temperature feedback mechanism.

Figure 3d shows histograms of the recorded temperatures over the 28 × 28 μm area at different reactor temperatures and with or without reaction gases. Maps of all measurements are shown in Figure S12. For comparison, the Gaussian solid lines reflect the estimated measurement uncertainties. The experiments under inert conditions all show an average temperature matching the reactor temperature, and variations are approximately as wide as expected based on the uncertainty. This confirms the validity of our local-calibration strategy, including the estimates of the temperature uncertainty. The experiments under the reaction conditions show temperatures exceeding the reactor temperature. The excess temperature is larger for a higher reactor temperature. This is expected as reaction rates, and hence heat generation, increase with temperature.^37^ Indeed, online mass spectroscopy confirms that a higher reactor temperature leads to a higher CH_4_ production (Figure 3e, see also Figure S6b). The experiments under the reaction conditions all show temperature variations wider than the measurement uncertainty. The magnitude of temperature variations is approximately constant with the reactor temperature. The trend of excess temperature and spatial variations as a function of reactor temperature is summarized in Figure 3f. The spatial variations in temperature with reaction gases could originate from several sources or a combination thereof, including (1) variations in density of active sites, (2) variations in catalyst activity per active site due to, for example, diffusion limitations, or (3) variations in heat dissipation. Further thermometry measurements and analyses could be used to determine, for example, spatial variations in apparent activation energy or the presence of diffusion barriers. The necessary measurement and analysis procedures would depend on the underlying causes of the temperature variations. Unraveling these causes may require correlated measurements using, for example, Raman spectroscopy for local concentrations of reaction intermediates or electron microscopy and X-ray techniques for local catalyst density or particle sizes. Such a combination of techniques could shine more in-depth light on structure–function relationships of catalyst materials, and the present work reveals the potential of luminescence thermometry as an additional *operando* technique allowing high-resolution temperature mapping to monitor catalyst activity on micrometer length scales.

### Considerations for Further Experiments

**Figure 4:**
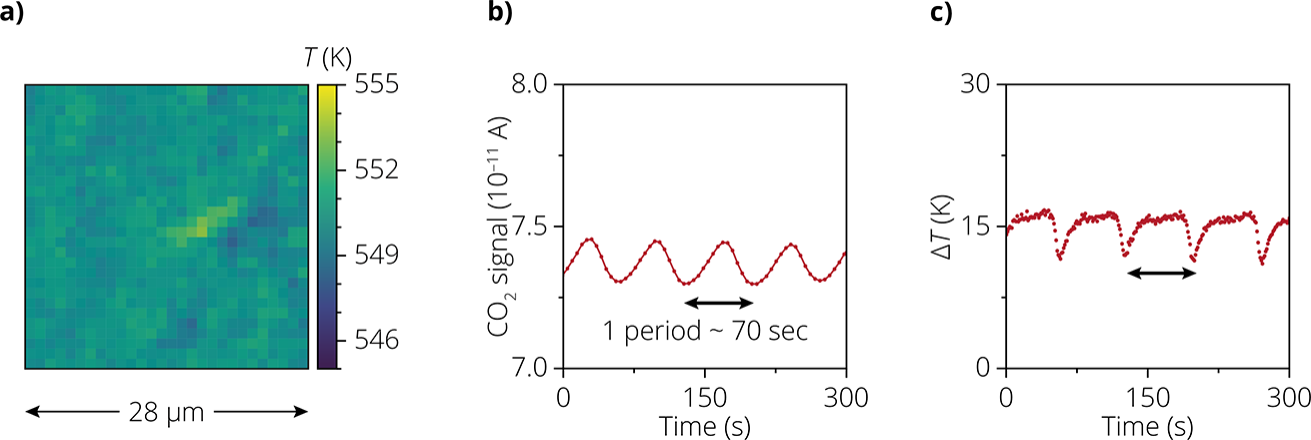
(a) 2D map of temperatures as in Figure 3b, but without the spatial drift correction. (b) Mass spectrometry (MS) trace for CO_2_ (*m*/*z* = 44), measured at the outlet of the Harrick cell. A clear periodicity of 70 s is observed, while the amplitude and shape of this graph are strongly affected by the slow instrument response of the mass spectrometer. (c) Single-pixel measurement to illustrate the oscillations in the local temperature relative to the reactor temperature (Δ*T*) due to the fluctuation in the CO_2_ flow.

The excess temperatures and spatial variations (Figure 3) are of the same order of magnitude as those of the potential artifacts originating from scattering, absorption, and background fluorescence. This highlights the importance of considering possible sources of systematic errors. For example, the unrealistic variations in apparent temperature obtained when we used external (Figure 1c) rather than local (Figure 2e) calibration are as wide as σ_T_ = 3.0 K for the experiment at 550 K under inert conditions. The local-calibration strategy resolves this issue. This strategy is, however, critically vulnerable to sample drift. As our experiments have micrometer resolution, the calibration measurements (data points in Figure 3a) must be performed on the same location within a micrometer. For our experiments under gas flow and at elevated temperatures, avoiding sample drift is difficult. We minimize drift by using a short integration time of 50 ms per pixel. This choice of integration time is possible because the thermometer particles are sufficiently bright, keeping the temperature uncertainties to within δ_T_ = 0.4 K. Despite the fast mapping, small drift corrections of 100–1000 nm are necessary to overlay the calibration measurements and subsequent experiments (see pages 16–18 in the Supporting Information). If we skip drift correction, minor but clearly correlated spatial variations appear in the temperature map recorded under inert conditions (Figure 4a), while only random noise is expected (compare to Figure 3b). Furthermore, in some experiments, we experienced periodic oscillations in the gas feed (Figure 4b), which we ascribe to instabilities in the mass-flow controller on the gas lines. This led to oscillations in the recorded temperature when measuring at a single location (Figure 4c). As collection of a total map takes a few seconds to minutes, depending on the map size and integration time per pixel, these oscillations could manifest as apparent spatial variations in temperature (Figure S14). Finally, we note that the determination of *absolute* temperatures is only as accurate as the temperature control during the calibration measurements. Any systematic deviation between the internal temperature reading of the sample cell and the actual temperature at the location of the sample affects the calibration model (eq 3) and, subsequently, the conversion of the LIR to temperature. We minimize this effect with reference experiments on the Harrick sample cell, in which we measure the absolute temperature at the location of the sample with an external thermocouple (*T*_reactor_) as a function of the set temperature of the cell (Figure S15).^38^ The sample temperatures calibrated in this way are used as inputs for the calibration model (Figure 3a).

## Conclusions

We have measured reaction-induced heating during CO_2_ methanation and revealed spatial variations in the catalytic activity. The emission maps were carefully characterized to correct for artifacts originating from wavelength-dependent absorption/scattering of the sample, background fluorescence, and sample drift. On average, we quantify excess temperatures of 6–25 K, which increases with increasing reactor set temperatures. Spatial variations of the readout temperature σ_T_ = 1.5 K exceed the expected temperature uncertainty of δ_T_ = 0.4 K, which is attributed to spatial variations in the catalytic reaction rate. Variations in temperature during catalysis could affect the selectivity of the reaction, cause thermal runaways, or could be used for more accurate simulations of chemical reactors. This shows that luminescence thermometry is a useful tool to map catalyst activity that can help to improve the design of catalysts and reactors.

## Experimental Methods

### Chemicals and Materials

All chemicals were used without further purification. The chemicals Y(NO_3_)_3_·6H_2_O, Nd(NO_3_)_3_·6H_2_O, and Ni(NO_3_)_2_·6H_2_O with a purity of 99.99% were purchased from Sigma-Aldrich. Titanium dioxide (TiO_2_ P25) was purchased from Acros Organics. Urea (99.5%) was purchased from Strem Chemicals. Ammonium oxalate (99.0%) was purchased from J.T. Baker Chemicals. Ammonia (25%) was purchased from Merck.

### Luminescent Thermometer Synthesis

The microcrystalline Y_2_O_3_:Nd^3+^ was prepared via a precipitation technique based on the works of Buijs et al.^39^ 4 mmol of RE(NO_3_)_3_ (RE = Y and Nd) was dissolved in 10 mL of ultrapure water (MQ), and 12 mmol of ammonium oxalate was dissolved in 50 mL of MQ. While stirring, the ammonium oxalate was added to the RE(NO_3_)_3_ solution causing RE oxalate to precipitate. The pH was checked by using a pH indicator strip. Ammonia was added until the pH was around 9, after which the solution was stirred for 1 h. Afterward, the solution was centrifuged (5000 rpm for 5 min) and decanted, and the precipitate was washed three times with water (MQ). The sample was dried overnight at 373 K and calcined at 1473 K in static air for 8 h by using a ramp of 5 K/min.

### Catalyst Synthesis

The nickel-based CO_2_ methanation catalyst, containing a TiO_2_ support, was prepared via a homogeneous deposition preparation, as described by Vogt et al.^40^ 1.6 mmol of Ni(NO_3_)_2_·6H_2_O and 51 mmol of urea were dissolved in 130 mL of water, to which 22.4 mmol of the TiO_2_ support (surface area of 41.6 m^2^/g and pore volume of 0.18 cm^3^/g) was added. This mixture was stirred by a mechanical stirring setup at 650 rpm and heated to 371 K using distilled water in a double-walled reaction vessel for 20 h. The mixture was left to cool while stirring for 1 h and then transferred to a beaker. The mixture was centrifuged for 3 min at 3500 rpm and washed with distilled water 5 times until the decanted supernatant was pH neutral. The precipitated slurry was first dried in a 333 K oven overnight and then placed in a 393 K oven again overnight.

### Mixture of Catalyst and Luminescent Thermometer

The as-prepared Y_2_O_3_:Nd^3+^ thermometer material and Ni/TiO_2_ catalyst material were mixed by grinding in a 1:4 mass ratio, both after grinding into a fine powder. The 1:4 mass ratio (or 1:3 volume ratio) was an increase of thermometer material compared to previous work and was used to ensure a sufficient amount of thermometer in every pixel.^7^ The resulting mixture was pelletized using 5 tons of force for 30 s (aiming for ø = 5 mm and thickness = 0.2 mm).

### Material Characterization

Scanning electron microscopy (SEM) images were obtained using a Phenom ProX microscope with an acceleration voltage of 10 kV. High-angle annular dark field scanning transmission electron microscopy (HAADF-STEM) images were obtained using a Talos F200x microscope with an acceleration voltage of 200 kV. X-ray diffraction (XRD) patterns were recorded with a Bruker D2 Phaser using Co Kα (λ = 1.79026 Å) radiation. The measured range in terms of 2θ was 20–80° with a step size of 0.02°. Inductively coupled plasma-optical emission spectroscopy (ICP-OES) measurements were conducted on a PerkinElmer ICP-OES Optima 8300. The Y_2_O_3_:Nd^3+^ samples were dissolved in concentrated nitric acid by sonication and diluted in a 5% nitric acid in MQ solution to obtain a concentration in the range of 0–1 ppm. The neodymium concentration was measured using the emission lines at 401.2, 406.1, and 430.3 nm, while the yttrium concentration was measured using the emission lines at 324.2, 360.1, and 371.0 nm. The dopant concentration of neodymium was calculated by using the average parts per million values obtained with measurements on these three emission lines. Photoluminescence decay curves were measured using an Ekspla NT342B OPO laser (λ_exc_ = 578–580 nm, 10 Hz), a Triax 550 monochromator (λ_em_ = 822–830 nm), and a Hamamatsu H7422 photomultiplier tube. The excitation and emission wavelengths were varied for the maximum signal intensity. A long-pass filter was used to block the excitation light, and the average count rate was kept at 1–50 counts per pulse to prevent detector saturation. The emission spectra for the calibration curve were measured using a Horiba Raman spectrometer with a 785 nm laser, 600 mm^–1^ grating, 50× objective (numerical aperture, NA = 0.5), and 1% laser power (0.48 mW or 10^5^ W/cm^2^). An edge filter was used to filter out the excitation light. The controlled heating in all of these experiments was performed in a Linkam TS1000 microscope stage without a gas flow.

### Catalytic Performance Measurements

The reaction chamber used was a Harrick Raman high-temperature chamber embedded with a Harrick Screen Set wafer (ø = 6.3 mm; pore size = 0.061 mm). The emission spectra were measured using a Horiba Raman spectrometer with a 785 nm laser, 50× objective (NA = 0.5), and 1% laser power (0.48 mW or 10^5^ W/cm^2^). The laser power used does not cause illumination-induced heating of the blackened sample (Figure S16). The catalysis experiments were performed using gases supplied by Linde (Ar, CO_2_, H_2_). The gas flows were controlled by Bronkhorst mass flow controllers (MFC). All experiments were performed at ambient pressure (ca. 101 kPa). The nickel nanoparticles were first reduced in a gas atmosphere of 20 mL/min H_2_ and 30 mL/min Ar while heating up to 685 K (*T*_set_ = 723 K) using a ramp of 5 K/min. The sample was cooled down back to 458 K (*T*_set_ = 473 K), and the gas atmosphere was varied between only Ar and a reaction mixture of 5 mL/min CO_2_, 20 mL/min H_2_, and 25 mL/min Ar. The temperature maps were constructed at reactor temperatures of 550, 595, and 640 K (*T*_set_ = 573, 623, and 673 K, respectively). At these temperatures, gas compositions were changed to study the exothermicity of the reaction (i.e., 0:0:50 or 5:20:25 for the CO_2_/H_2_ introduction experiments). The mapping experiments were performed at stable CH_4_ production. Time-resolved temperature measurements were performed during switching of the gas atmosphere. The gas composition was monitored with online mass spectrometry (MS) on an Omni Star GSD 320 O2 Analytical system (Pfeiffer Vacuum). The inlet was heated to 423 K.

## Supporting Information Available

Additional information on the characterization of materials (XRD/TEM/SEM), calculations of the LIR, relative sensitivity, cross-relaxation strength, efficiencies, temperature uncertainties, and local calibration procedure (PDF)

## References

[ref1] Buurmans I. L. C.; Weckhuysen B. M. Heterogeneities of individual catalyst particles in space and time as monitored by spectroscopy. Nat. Chem. 2012, 4, 873–886. 10.1038/nchem.1478. 23089861

[ref2] Pessoa A. R.; Galindo J. A. O; Serge-Correales Y. E.; Amaral A. M.; Ribeiro S. J. L.; Menezes L. 2D Thermal Maps Using Hyperspectral Scanning of Single Upconverting Microcrystals: Experimental Artifacts and Image Processing. ACS Appl. Mater. Interface 2022, 14, 38311–38319. 10.1021/acsami.2c08709. 35969002

[ref3] Piotrowski W.; Dalipi L.; Elzbieciak-Piecka K.; Bednarkiewicz A.; Fond B.; Marciniak L. Self-Referenced Temperature Imaging with Dual Light Emitting Diode Excitation and Single-Band Emission of AVO_4_:Eu^3+^ (A = Y, La, Lu, Gd) Nanophosphors. Adv. Photonics Res. 2022, 3, 2100139 10.1002/adpr.202100139.

[ref4] Kočí P.; Štěpánek F.; Kubíček M.; Marek M. Modelling of micro/nano-scale concentration and temperature gradients in porous supported catalysts. Chem. Eng. Sci. 2007, 62, 5380–5385. 10.1016/j.ces.2006.12.033.

[ref5] Hwang S.; Smith R. Optimum reactor design in methanation processes with nonuniform catalysts. Chem. Eng. Commun. 2008, 196, 616–642. 10.1080/00986440802484465.

[ref6] Hartman T.; Geitenbeek R. G.; Whiting G. T.; Weckhuysen B. M. Operando monitoring of temperature and active species at the single catalyst particle level. Nat. Catal. 2019, 2, 986–996. 10.1038/s41929-019-0352-1.

[ref7] Geitenbeek R. G.; Nieuwelink A.-E.; Jacobs T. S.; Salzmann B. B. V.; Goetze J.; Meijerink A.; Weckhuysen B. M. In Situ Luminescence Thermometry to Locally Measure Temperature Gradients during Catalytic Reactions. ACS Catal. 2018, 8, 2397–2401. 10.1021/acscatal.7b04154. 29527404 PMC5839602

[ref8] Jaque D.; Vetrone F. Luminescence nanothermometry. Nanoscale 2012, 4, 4301–4326. 10.1039/c2nr30764b. 22751683

[ref9] Xuan G.; Mirko E.; Rodrigues S. J.; Vorhauer-Huget N.; Christian L.; Fond B. Multi-point temperature measurements in packed beds using phosphor thermometry and ray tracing simulations. Particuology 2024, 85, 77–88. 10.1016/j.partic.2023.03.015.

[ref10] Hartman T.; Geitenbeek R. G.; Wondergem C. S.; Van Der Stam W.; Weckhuysen B. M. Operando Nanoscale Sensors in Catalysis: All Eyes on Catalyst Particles. ACS Nano 2020, 14, 3725–3735. 10.1021/acsnano.9b09834. 32307982 PMC7199205

[ref11] Monai M.; Jenkinson K.; Melcherts A. E. M.; Louwen J. P.; Irmak E. A.; van Aert S.; Altzantzis T.; Vogt C.; van der Stam W.; Duchon T.; Smíd B.; Groeneveld E.; Berben P.; Bals S.; Weckhuysen B. M. Restructuring of titanium oxide overlayers over nickel nanoparticles during catalysis. Science 2023, 380, 644–651. 10.1126/science.adf6984. 37167405

[ref12] Rabouw F. T.; Prins T. P.; Villanueva-Delgado P.; Castelijns M.; Geitenbeek R. G.; Meijerink A. Quenching Pathways in NaYF_4_:Er^3+^, Yb^3+^ Upconversion Nanocrystals. ACS Nano 2018, 12, 4812–4823. 10.1021/acsnano.8b01545. 29648802 PMC5968434

[ref13] Runowski M.; Wózny P.; Stopikowska N.; Martín I. R.; Lavín V.; Lis S. Luminescent nanothermometer operating at very high temperature-sensing up to 1000 K with upconverting nanoparticles (Yb^3+^/Tm^3+^). ACS Appl. Mater. Interface 2020, 12, 43933–43941. 10.1021/acsami.0c13011. PMC7660569 32869638

[ref14] Brites C. D. S.; Balabhadra S.; Carlos L. D. Lanthanide-Based Thermometers: At the Cutting-Edge of Luminescence Thermometry. Adv. Opt. Mater. 2019, 7, 1801239 10.1002/adom.201801239.

[ref15] Yu D.; Li H.; Zhang D.; Zhang Q.; Meijerink A.; Suta M. One ion to catch them all: Targeted high-precision Boltzmann thermometry over a wide temperature range with Gd^3+^. Light Sci. Appl. 2021, 10, 4–15. 10.1038/s41377-021-00677-5. 34811347 PMC8608900

[ref16] Suta M.; Meijerink A. A Theoretical Framework for Ratiometric Single Ion Luminescent Thermometers—Thermodynamic and Kinetic Guidelines for Optimized Performance. Adv. Theory Simul. 2020, 3, 2000176 10.1002/adts.202000176.

[ref17] Terlingen B. J. P.; Arens T.; van Swieten T. P.; Rabouw F. T.; Prins T.; de Beer M. M.; Meijerink A.; Ahr M.; Hutter E. M.; van Lare C.; Weckhuysen B. M. Bifunctional Europium for Operando Catalyst Thermometry in an Exothermic Chemical Reaction. Angew. Chem., Int. Ed. 2022, 61, e202211991 10.1002/anie.202211991. PMC10099702 36328981

[ref18] Brites C. D. S.; Lima P. P.; Silva N. J. O.; Millán A.; Amaral V. S.; Palacio F.; Carlos L. D. Thermometry at the nanoscale. Nanoscale 2012, 4, 4799–4829. 10.1039/c2nr30663h. 22763389

[ref19] van Swieten T. P.; van Omme T.; van den Heuvel D. J.; Vonk S. J. W.; Spruit R. G.; Meirer F.; Garza H. P.; Weckhuysen B. M.; Meijerink A.; Rabouw F. T.; Geitenbeek R. G. Mapping Elevated Temperatures with a Micrometer Resolution Using the Luminescence of Chemically Stable Upconversion Nanoparticles. ACS Appl. Nano Mater. 2021, 4, 4208–4215. 10.1021/acsanm.1c00657. 34085030 PMC8162758

[ref20] Ximendes E. C.; Santos W. Q.; Rocha U.; Kagola U. K.; Sanz-Rodríguez F.; Fernández N.; Gouveia-Neto A.; Bravo D.; Domingo A. M.; Rosal B.; Brites C. D. S.; Carlos L. D.; Jaque D.; Jacinto C. Unveiling in Vivo Subcutaneous Thermal Dynamics by Infrared Luminescent Nanothermometers. Nano Lett. 2016, 16, 1695–1703. 10.1021/acs.nanolett.5b04611. 26845418

[ref21] Italiano C.; Llorca J.; Pino L.; Ferraro M.; Antonucci V.; Vita A. CO and CO_2_ methanation over Ni catalysts supported on CeO_2_, Al_2_O_3_ and Y_2_O_3_ oxides. Appl. Catal. B Environ. 2020, 264, 118494 10.1016/j.apcatb.2019.118494.

[ref22] Li Y.; Men Y.; Liu S.; Wang J.; Wang K.; Tang Y.; An W.; Pan X.; Li L. Remarkably efficient and stable Ni/Y_2_O_3_ catalysts for CO_2_ methanation: Effect of citric acid addition. Appl. Catal. B Environ. 2021, 293, 120206 10.1016/j.apcatb.2021.120206.

[ref23] Tada S.; Shimizu T.; Kameyama H.; Haneda T.; Kikuchi R. Ni/CeO_2_ catalysts with high CO_2_ methanation activity and high CH_4_ selectivity at low temperatures. Int. J. Hydrogen Energy 2012, 37, 5527–5531. 10.1016/j.ijhydene.2011.12.122.

[ref24] Ye R.-P.; Ding J.; Gong W.; Argyle M. D.; Zhong Q.; Wang Y.; Russell C. K.; Xu Z.; Russell A. G.; Li Q.; Fan M.; Yao Y.-G. CO_2_ hydrogenation to high-value products via heterogeneous catalysis. Nat. Commun. 2019, 10, 5698 10.1038/s41467-019-13638-9. 31836709 PMC6910949

[ref25] Kolesnikov I. E.; Kalinichev A. A.; Kurochkin M. A.; Mamonova D. V.; Kolesnikov E. Y.; Lähderanta E.; Mikhailov M. D. Bifunctional heater-thermometer Nd^3+^-doped nanoparticles with multiple temperature sensing parameters. Nanotechnology 2019, 30, 145501 10.1088/1361-6528/aafcb8. 30625447

[ref26] Geitenbeek R. G.; Salzmann B. B. V.; Nieuwelink A. E.; Meijerink A.; Weckhuysen B. M. Chemically and thermally stable lanthanide-doped Y_2_O_3_ nanoparticles for remote temperature sensing in catalytic environments. Chem. Eng. Sci. 2019, 198, 235–240. 10.1016/j.ces.2018.10.004.

[ref27] Suta M.; Antic Z.; Djordevic V.; Kuzman S.; Dramicanin M. D.; Meijerink A. Making Nd^3+^ a Sensitive Luminescent Thermometer for Physiological Temperatures — An Account of Pitfalls in Boltzmann Thermometry. Nanomaterials 2020, 10, 543 10.3390/nano10030543. 32197319 PMC7153599

[ref28] van Swieten T. P.; Steenhoff J. M.; Vlasblom A.; de Berg R.; Mattern S. P.; Rabouw F. T.; Suta M.; Meijerink A. Extending the dynamic temperature range of Boltzmann thermometers. Light Sci. Appl. 2022, 11, 434 10.1038/s41377-022-01028-8. PMC9732288 36481747

[ref29] Vollmer I.; Yarulina I.; Kapteijn F.; Gascon J. Progress in Developing a Structure-Activity Relationship for the Direct Aromatization of Methane. ChemCatChem. 2019, 11, 39–52. 10.1002/cctc.201800880.

[ref30] Aramouni N. A. K.; Touma J. G.; Tarboush B. A.; Zeaiter J.; Ahmad M. N. Catalyst design for dry reforming of methane: Analysis review. Renew. Sustain. Energy Rev. 2018, 82, 2570–2585. 10.1016/j.rser.2017.09.076.

[ref31] Carnall W. T.; Crosswhite H.; Crosswhite H. M. Energy level Structure and Transition probabilities in the Spectra of Trivalent Lanthanides in LaF_3_; Argonne National Laboratory: 1978.

[ref32] van Swieten T. P.; Yu D.; Yu T.; Vonk S. J. W.; Suta M.; Zhang Q.; Meijerink A.; Rabouw F. T. Ho^3+^-Based Luminescent Thermometer for Sensitive Sensing over a Wide Temperature Range. Adv. Opt. Mater. 2021, 9, 2001518 10.1002/adom.202001518.

[ref33] Rabouw F. T.; Den Hartog S. A.; Senden T.; Meijerink A. Photonic effects on the Förster resonance energy transfer efficiency. Nat. Commun. 2014, 5, 3610 10.1038/ncomms4610. 24694758

[ref34] Vonk S. J. W.; van Swieten T. P.; Cocina A.; Rabouw F. T. Photonic Artifacts in Ratiometric Luminescence Nanothermometry. Nano Lett. 2023, 23, 6560–6566. 10.1021/acs.nanolett.3c01602. 37450686 PMC10375589

[ref35] Kopyscinski J.; Schildhauer T. J.; Vogel F.; Biollaz S. M. A.; Wokaun A. Applying spatially resolved concentration and temperature measurements in a catalytic plate reactor for the kinetic study of CO methanation. J. Catal. 2010, 271, 262–279. 10.1016/j.jcat.2010.02.008.

[ref36] Mutschler R.; Moioli E.; Zhao K.; Lombardo L.; Oveisi E.; Porta A.; Falbo L.; Visconti C. G.; Lietti L.; Züttel A. Imaging Catalysis: Operando Investigation of the CO_2_ Hydrogenation Reaction Dynamics by Means of Infrared Thermography. ACS Catal. 2020, 10, 1721–1730. 10.1021/acscatal.9b04475.

[ref37] Vogt C.; Groeneveld E.; Kamsma G.; Nachtegaal M.; Lu L.; Kiely C. J.; Berben P. H.; Meirer F.; Weckhuysen B. M. Unravelling structure sensitivity in CO_2_ hydrogenation over nickel. Nat. Catal. 2018, 1, 127–134. 10.1038/s41929-017-0016-y.

[ref38] Li H.; Rivallan M.; Thibault-Starzyk F.; Travert A.; Meunier F. C. Effective bulk and surface temperatures of the catalyst bed of FT-IR cells used for in situ and operando studies. Phys. Chem. Chem. Phys. 2013, 15, 7321–7327. 10.1039/c3cp50442e. 23576134

[ref39] Buijs M.; Meyerink A.; Blasse G. Energy Transfer between Eu^3+^ Ions in a Lattice with Two Different Crystallographic Sites: Y_2_O_3_:Eu^3+^, Gd_2_O_3_:Eu^3+^ and Eu_2_O_3_. J. Lumin. 1987, 37, 9–20. 10.1016/0022-2313(87)90177-3.

[ref40] Vogt C.; Monai M.; Sterk E. B.; Palle J.; Melcherts A. E. M.; Zijlstra B.; Groeneveld E.; Berben P. H.; Boereboom J. M.; Hensen E. J. M.; Meirer F.; Filot I. A. W.; Weckhuysen B. M. Understanding carbon dioxide activation and carbon–carbon coupling over nickel. Nat. Commun. 2019, 10, 5330 10.1038/s41467-019-12858-3. 31767838 PMC6877608

