# Supplementary material for: Mapping Temperature Heterogeneities during Catalytic CO2 Methanation with Operando Luminescence Thermometry
Source: ACS Nano. 2023 Oct 5;17(20):20053–61. doi: 10.1021/acsnano.3c05622 (PMC10604088; doi:10.1021/acsnano.3c05622)
Supplement: Supplementary file 1 — nn3c05622_si_001.pdf [file nn3c05622_si_001.pdf]

Supporting Information for:

Mapping Temperature Heterogeneities during Catalytic CO_2 Methanation with *Operando* Luminescence Thermometry

Thimo S. Jacobs [a)], *Thomas P. van Swieten* [b)], *Sander J. W. Vonk* [a,c)], *Isa P. Bosman* [a)], *Angela E. M. Melcherts* [a)], *Bas C. Janssen* [a)], *Joris C. L. Janssens* [a)], *Matteo Monai* [a)], *Andries Meijerink* [b)], *Freddy T. Rabouw* [a,c),*], *Ward van der Stam* [a),*], *and Bert M. Weckhuysen* [a),*]

a) Inorganic Chemistry and Catalysis, Debye Institute for Nanomaterials Science and Institute for Sustainable and Circular Chemistry, Utrecht University, Universiteitsweg 99, 3584 CG Utrecht, The Netherlands

b) Condensed Matter and Interfaces, Debye Institute for Nanomaterials Science, Utrecht University, Princetonplein 1, 3584 CC Utrecht, The Netherlands

c) Soft Condensed Matter and Biophysics, Debye Institute for Nanomaterials Science, Utrecht University, Princetonplein 1, 3584 CC Utrecht, The Netherlands

* Authors to whom correspondence should be addressed, electronic mail: f.t.rabouw@uu.nl, w.vanderstam@uu.nl and b.m.weckhuysen@uu.nl

(22 pages including 16 figures)

Characterization of the materials

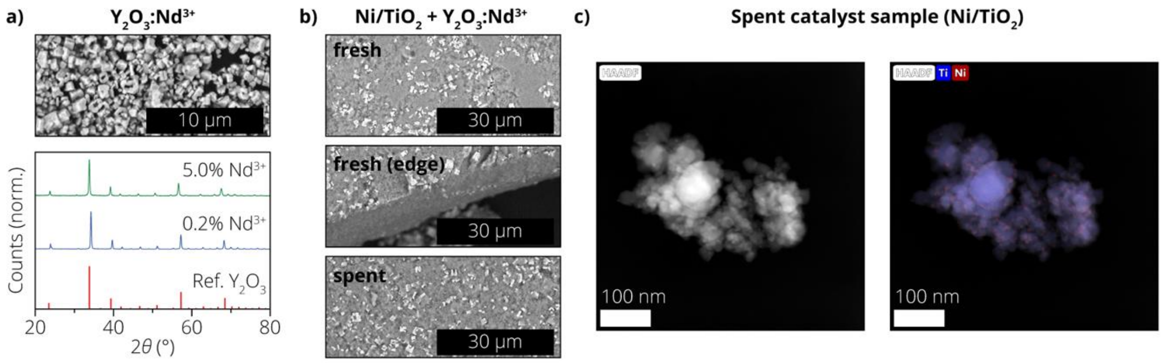

Figure S1. Characterization of the materials described in the main text of the article. a) Scanning Electron Microscopy (SEM, top) and X-ray Diffraction (XRD, bottom) of the microcrystalline $Y_2O_3:Nd^{3+}$. The SEM image highlights the cubic crystal structure (Ia-3 space group) of the Y_2O_3. Scalebar in the SEM image represents 10 µm. The X-ray diffractograms show the crystallographic properties of $Y_2O_3:Nd^{3+}$ with 0.2% (blue line) and 5% (green line) of Nd^{3+}. The reference of Y_2O_3 matches with the diffractograms (red bars, database no. 00-041-1105). b) SEM images of the mixture of Ni/TiO_2 and $Y_2O_3:Nd^{3+}$, with the fresh sample (top), the edge of the fresh pellet (middle), and the spent sample (bottom). All scalebars represent 30 µm. The white particles are the microcrystalline $Y_2O_3:Nd^{3+}$, and the grey material is the TiO_2 support. The nickel nanoparticles are too small to be visible with SEM. c) Transmission Electron Microscopy (TEM) image of the spent sample containing Ni and TiO_2 after the catalytic CO_2 methanation reaction. The Y_2O_3 was not found, probably due to the preparation of the TEM grid. Electron Dispersive X-Ray (EDX) images were added to highlight the position of certain elements. Scalebar in the TEM image represents 100 nm.

Table S1. Inductively Coupled Plasma Optical Emission Spectroscopy (ICP-OES) results of elemental analysis for dopant concentrations of Nd^{3+} in Y_2O_3. The neodymium lines used were 401.225, 406.109, and 430.358 nm and the results from these lines were averaged. The yttrium lines used were 324.227, 360.073, and 371.029 nm and the results from these lines were averaged. * = the Nd^{3+} content in the sample with the targeted Nd^{3+} concentration of 0.2% was below the detection limit of the ICP-OES.

Sample	Target Nd^{3+} conc. (%)	Measured Nd^{3+} conc. (%)
$Y_2O_3:Nd^{3+}$ (0.2%)	0.2	<0.6%*
$Y_2O_3:Nd^{3+}$ (1%)	1.0	1.10% ± 0.14
$Y_2O_3:Nd^{3+}$ (1.3%)	1.3	1.15% ± 0.11
$Y_2O_3:Nd^{3+}$ (2%)	2.0	3.35% ± 0.17
$Y_2O_3:Nd^{3+}$ (5%)	5.0	4.91% ± 0.37

Calculating the LIR and constructing the calibration curve

The luminescence intensity ratio LIR at temperatures of $T > 400$ K was fitted to a Boltzmann model:[1]

$$\mathrm{LIR} = \frac{I_2}{I_1} = C\mathrm{e}^{-\Delta E/k_\mathrm{B}T} \qquad (1)$$

Here, I_i is the integrated intensity of emission line i, C is a prefactor including the spontaneous emission rates and degeneracies of the excited states, ΔE is the energy difference between the thermally coupled excited states, k_B is the Boltzmann constant, and T the temperature. Integration ranges of 795–860 nm and 860–960 nm were used for I_2 and I_1, respectively (Figure S2a). At temperatures above 963 K, the spectra were first corrected for black-body radiation (Figure S2b). The slope of ln(LIR) versus $1/T$ in the calibration curve (Figure 1c in the main text) is consistent with a value of $\Delta E = 996\ \mathrm{cm}^{-1}$.

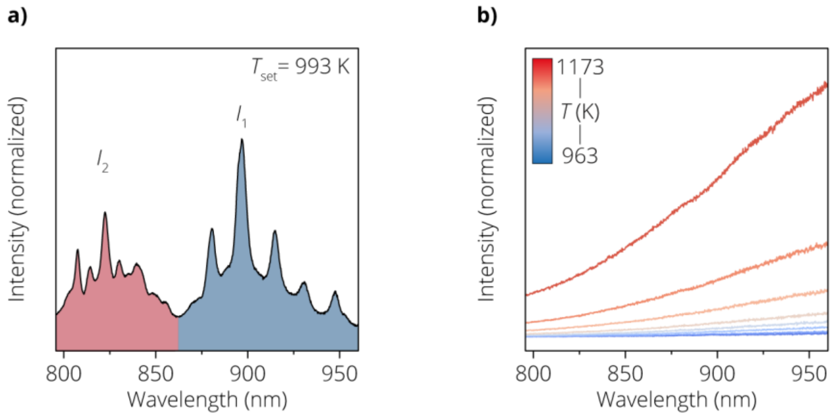

Figure S2. a) Emission spectrum of Y_2O_3 doped with 2% of neodymium at 993 K (corrected for black-body radiation), excited with the 785 nm laser of the Raman microscope. The luminescence intensity ratio is calculated by dividing the red area (I_2, $^4F_{5/2}$ to $^4I_{9/2}$ electronic transition) over the blue area (I_1, $^4F_{3/2}$ to $^4I_{9/2}$ electronic transition). b) Black-body radiation measured using the same settings as in a), but without laser excitation. The spectra were collected from 963 K (blue) to 1173 K (red), with intervals of 30 K.

Calculating the cross-relaxation strength

The cross-relaxation rate k_x scales strongly with the distance R between a Nd^{3+} ion in the excited state and a Nd^{3+} in the ground state (as shown in Figure 1f), as $k_x = C_x R^{-6}$. Substitutional doping (Nd^{3+} for Y^{3+}) introduces optically active dopants at discrete lattice sites, but with varying numbers of neighboring dopants in the nearest neighbor shell, next-nearest neighbor shell, etc. To model the photoluminescence decay curve at a particular Nd^{3+} concentration, we assume random substitutional doping.[2] We take into account all distances to neighbors from the two types of lattice sites in the Y_2O_3 crystal, and assume equal rate constants for radiative decay k_r and cross-relaxation C_x for Nd^{3+} on either site. This produces the following model function for the photoluminescence decay $I(t)$ as a function of the doping concentration ϕ:

$$I(t) = I_0 X(C_x, \phi, t)\mathrm{e}^{-k(T)t} + y_0 \qquad \text{with} \tag{2}$$

$$X(C_x, \phi, t) = \frac{3}{4}\prod(1 - \phi + \phi \mathrm{e}^{-C_x t/R_i^6})^{n_i} + \frac{1}{4}\prod(1 - \phi + \phi \mathrm{e}^{-C_x t/R_i^{*6}})^{n_i^*}$$

Here, I_0 is the amplitude of the curve, k is the intrinsic decay rate of a Nd^{3+} dopant (i.e., excluding ion–ion interactions) and y_0 is the background intensity. N_i is the number of cations at a discrete distance R_i from a C_2 site (24d in Wyckoff notation) of the Y_2O_3 host crystal, and n_i^*, R_i^* are and the same but for an S_6 site (8b) (Figure S3). Our global fit procedure optimizes k and C_x simultaneously. Since the rates of cross-relaxation are approximately constant over the whole temperature range, the C_x from the global fit approach on the room-temperature data can be used to describe the cross-relaxation interactions between Nd^{3+} ions at all temperatures.

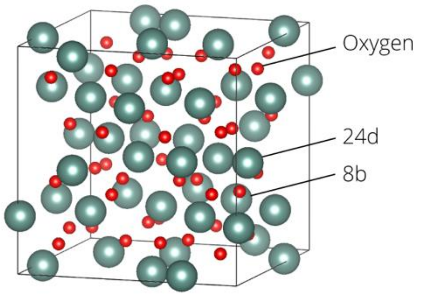

Figure S3. Schematic representation of the unit cell of Y_2O_3 (space group Ia-3), obtained from the VESTA software. The oxygen (red), 8b (grey/green) and 24d (green) sites are highlighted.

Calculating the efficiencies

To estimate the Nd^{3+} emission efficiencies as a function of temperature and doping concentration, we first characterize the temperature-independent radiative decay rate k_r and the temperature-dependent nonradiative decay rate k_{nr}. Figure S4a shows the decay traces of the thermometer at a low Nd^{3+} concentration (0.2%) at 293 K and 1023 K. These decay curves and those at a range of other temperature between 293 and 1173 K were fitted to a single exponent. The inset of Figure S4a shows the increase of the total decay rate with temperature, which is caused by thermal quenching.[3,4] This increase in the non-radiative rate at constant radiative rate leads to a lowered signal intensity of the lanthanide emission at temperatures above 900 K, which is undesired for precise temperature readouts.[5,6] We exclude that this quenching is due to multi-phonon relaxation, which is the coupling of host lattice phonons leading to relaxation from the thermally coupled excited states to the underlying energy level ($^4F_{3/2} \rightarrow {}^4I_{15/2}$).[5] Multi-phonon relaxation is highly improbable as a non-radiative decay pathway in Y_2O_3, since the energy gap between the $^4F_{3/2}$ and $^4I_{15/2}$ levels is ~5500 cm^{-1}, requiring nine maximum energy phonons to bridge that gap (maximum phonon energy of Y_2O_3 is around 600 cm^{-1}).[5,7,8] Indeed, thermal quenching sets in at different temperatures for three similar rare earth oxides: Gd_2O_3, Y_2O_3, and Sc_2O_3 (Figure S4b). These host materials have comparable phonon energies (Figure S4c), so similar multi-photon relaxation rates are expected. The quenching temperatures are different, so the quenching process is not multi-phonon relaxation.

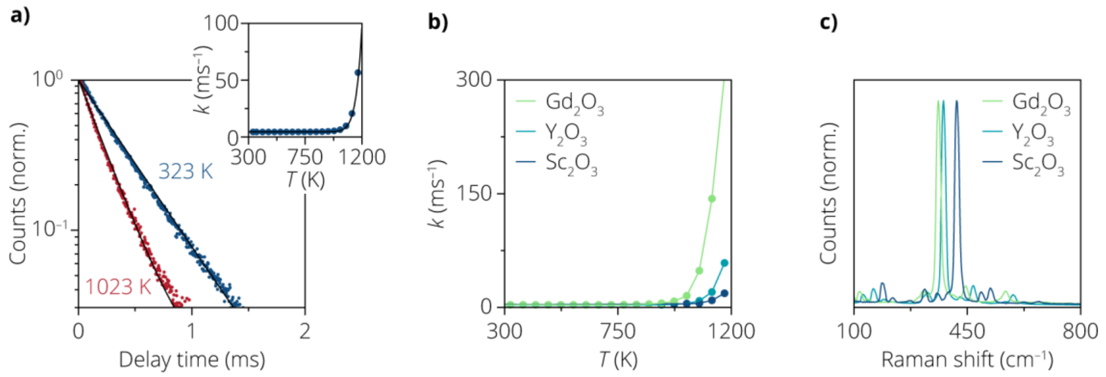

Figure S4. a) Photoluminescence decay curves (measured at 822–831 nm, upon excitation at 579–581 nm) of the thermally coupled $^4F_{5/2}$/$^4F_{3/2}$ energy levels of the 0.2%-doped Nd^{3+} sample at 323 K (blue) and 1023 K (red). The solid black lines are fitted to a single-exponential decay model. The inset shows the decay rates obtained from fits between 293 K and 1173 K. The black line is a fit to a model for the temperature-dependent cross-over via a charge transfer state. b) Decay rates of the $^4F_{3/2}$ level for 0.2% of neodymium in different host crystal lattices, gadolinium oxide (green), yttrium oxide (light blue) and scandium oxide (dark blue) at temperatures of 300–1200 K. The ionic radius order is (coordination number = 6): Gd^{3+} (0.938 Å) > Y^{3+} (0.9 Å) > Sc^{3+} (0.745 Å), which follows the inverse trend of the onset temperature at which non-radiative decay rates become dominant. Multi-phonon relaxation can be excluded as the dominant non-radiative decay pathway based on this data, since the onset temperature would be equal in all host crystal lattices (phonon energy is comparable in all oxide lattices. c) Phonon energies for the crystal oxide lattices, measured with the Raman microscope (λ = 638 nm). The phonon energies with the highest intensities are at 360, 376 and 416 cm^{-1} for Gd_2O_3, Y_2O_3 and Sc_2O_3, respectively.[9]

We fit the temperature-dependent decay rates to a constant term plus a model of non-radiative decay via cross-over to the ground state via a charge-transfer state:

$$k(T) = k_{\mathrm{const}} + k_{\mathrm{nr}}^{(0)} \mathrm{e}^{-E_{\mathrm{a}}/k_{\mathrm{B}}T} \tag{3}$$

We estimate the radiative decay rate k_{r} by multiplying the constant decay term k_{const} with the radiative efficiency of 0.2% Nd^{3+} doping at room temperature (Figure S5a). This value of k_{r} is used in the global fit of Figure 1d of the main text to find the cross-relaxation strength C_{x}. The results from the global fit can be used to calculate the probabilities of radiative decay (η_{r}), nonradiative decay (η_{nr}) and cross-relaxation (η_{x}) from the emitting levels of Nd^{3+}, as a function of temperature T and doping concentration ϕ:[3]

$$\eta_{\mathrm{r}} = k_{\mathrm{r}} \int_0^{\infty} \mathrm{e}^{-[k_{\mathrm{r}}t + k_{\mathrm{nr}}(T)t]} X(C_{\mathrm{x}}, \phi, t)\mathrm{d}t \tag{4}$$

$$\eta_{\mathrm{nr}} = k_{\mathrm{nr}}(T) \int_0^{\infty} \mathrm{e}^{-[k_{\mathrm{r}}t + k_{\mathrm{nr}}(T)t]} X(C_{\mathrm{x}}, \phi, t)\mathrm{d}t \tag{5}$$

$$\eta_{\mathrm{x}} = 1 - \eta_{\mathrm{nr}} - \eta_{\mathrm{r}} \tag{6}$$

Figure S5 shows the calculated probabilities of the different decay pathways. The efficiency of radiative decay and the probability of cross-relaxation decrease with temperature, while the probability of nonradiative decay increases. The increase in concentration results in an increased probability of cross-relaxation, while the probabilities of the radiative and nonradiative pathways decrease.

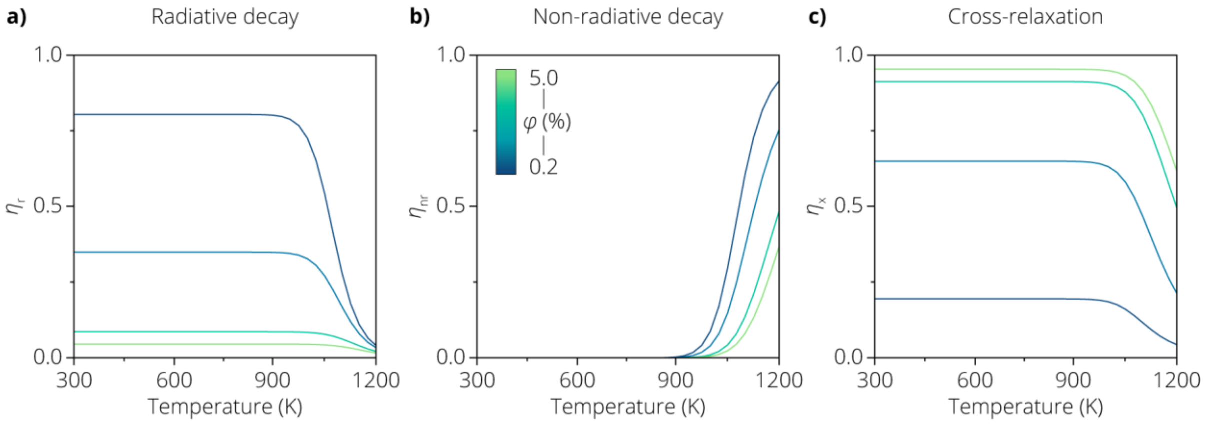

Figure S5. The probabilities of a) radiative decay (η_r), b) non-radiative decay (η_{nr}) and c) cross-relaxation (η_x) from the thermally coupled energy levels of Nd^{3+} as a function of *T*. Differently colored lines denote calculations for different Nd^{3+} doping concentrations of 0.2, 1.1, 3.4, and 4.9%, as indicated in panel b.

Calculating the relative temperature uncertainty

The expected uncertainty in temperature σ_T for a Boltzmann thermometer with Poissonian counting noise on the number of photon counts in the two emission bands is:[3]

$$\sigma_T(\phi) = \frac{1}{S_\mathrm{r}}\sqrt{\frac{1}{\phi}}\sqrt{\frac{1}{I_1}+\frac{1}{I_2}} = A\frac{1}{S_\mathrm{r}}\sqrt{\frac{1}{\phi}}\sqrt{\frac{1}{\eta_\mathrm{r}}}\sqrt{\frac{1}{k_{\mathrm{r}1}f_1}+\frac{1}{k_{\mathrm{r}2}f_2}} \tag{7}$$

which depends on the Boltzmann populations f_i of the emitting states i = 1,2:

$$f_1 = \frac{g_1}{g_1+g_2\mathrm{e}^{-\Delta E/k_\mathrm{B}T}} \text{ and } f_2 = \frac{g_2\mathrm{e}^{-\Delta E/k_\mathrm{B}T}}{g_1+g_2\mathrm{e}^{-\Delta E/k_\mathrm{B}T}}$$

Here, g_i is the degeneracy of level i, $k_{\mathrm{r}i}$ is the rate of radiative transition from emitting level i to the ground state, and A is a prefactor that depends on detection efficiency, measurement duration, etc. Any effect of wavelength-dependent scattering could be taken into account by adjusting $k_{\mathrm{r}i}$, but for our calculations we assume that $k_{\mathrm{r}2}/k_{\mathrm{r}1} = C$, where C is the prefactor in the calibration curve (Eq. S1).

The normalization of the temperature uncertainty, yielding the graph in Figure 1g, was performed by dividing all the calculated temperature uncertainties at a specific temperature (*e.g.*, 300 K) by the minimum temperature uncertainty at that temperature. We could therefore determine the optimal doping concentration at every temperature.

Introducing CO_2 into the gas atmosphere

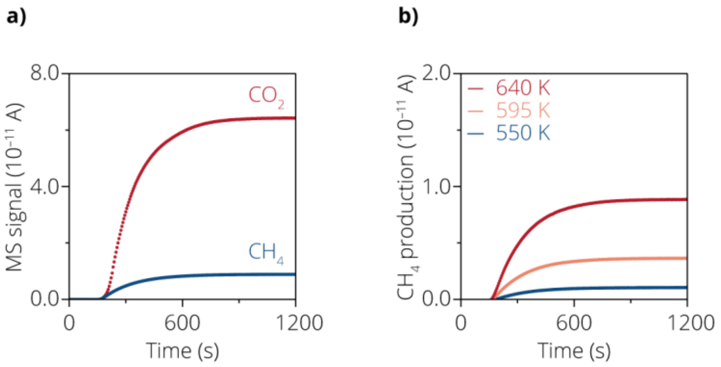

Figure S6. a) Mass Spectrometry (MS) traces for CO_2 (red, m/z = 44) and CH_4 (blue, m/z = 15) at a reactor temperature of 640 K, after the introduction of CO_2 and H_2 into the Harrick cell. b) MS traces for CH_4 at reactor temperatures of 550, 595 and 640 K, respectively.

Temperature variations with the calibration using a single value for *C*

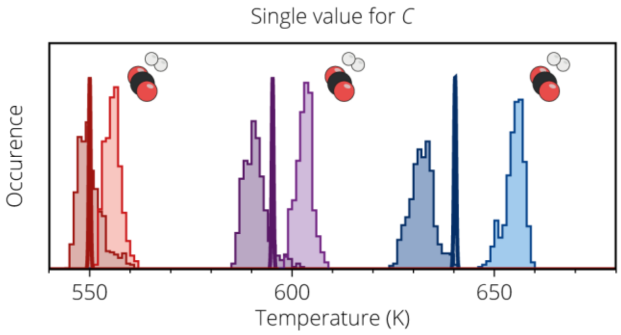

Figure S7. Histograms of the local temperatures recorded at different reactor temperatures (550, 595 and 640 K, going from red to blue). The histograms with the CO_2 and H_2 molecules are measured during the catalytic reaction. The Gaussian solid lines reflect the measurement uncertainties at the reactor temperatures.

Color change of the studied material before and after CO_2 methanation

Upon reducing the nickel hydroxide to nickel oxide/metallic nickel, the catalyst material changes color from green to black (Figure S8). During the CO_2 methanation experiment (after reduction), the sample remains black.

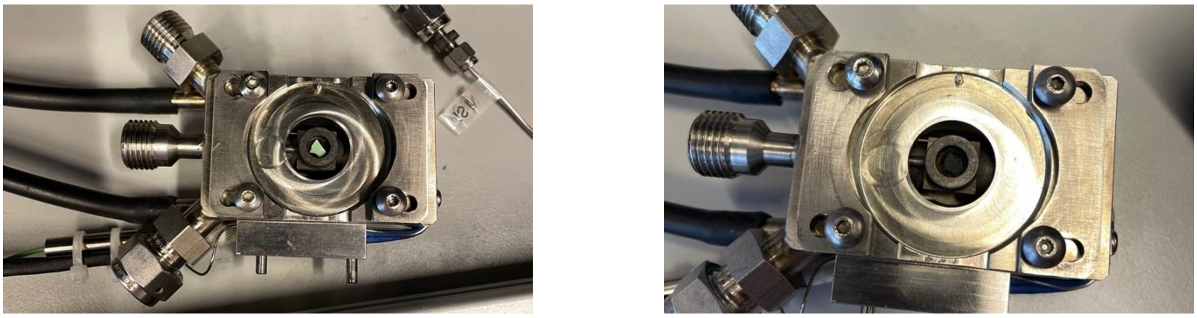

Figure S8. Left: mixture of Ni/TiO_2 and $Y_2O_3:Nd^{3+}$ before the catalytic CO_2 methanation, right: same material, but after the reduction.

Variations in LIR on a sample containing only $Y_2O_3:Nd^{3+}$

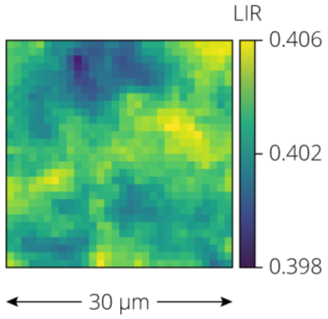

Figure S9. Two-dimensional (2D) map of the measured luminescence intensity ratios (LIR) for a sample containing only $Y_2O_3:Nd^{3+}$. The emission was measured under Ar atmosphere on a 30×30 µm area at a reactor temperature of 640 K. Integration time per pixel was 0.05 s. The variations in LIR in this map exceed the variations based on photon-counting noise.

Variations in background on a sample containing catalyst and undoped Y_2O_3

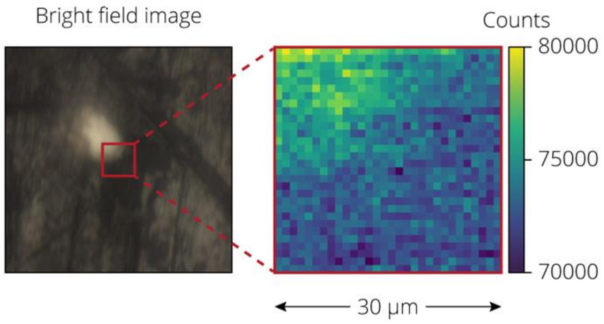

Figure S10. 2D map of the total background fluorescence counts between 795–960 nm. The background signal was measured at room temperature on a 30x30 µm area, without gas flow, on a sample containing catalyst and Y_2O_3 (without Nd^{3+}). Integration time per pixel was 0.05 s.

Room temperature measurement of variations in LIR

At evelated temperatures, preferential paths for the (colder) gas flow through the catalyst powder, which could lead to variations in the cooling of the sample and result in variations in LIR. We therefore performed a measurement at room temperature on the reduced (blackened) sample, without a gas flow. The result of the measurement at room temperature is shown in Figure S11, which shows that even without a gas flow and without heating, there are still variations in LIR. The uncertainty in LIR due to random noise was more than an order of magnitude smaller than the measured variations in LIR (compare 10^{-3} variations in Figure S11a versus 10^{-5}–10^{-4} errors in Figure S11b).

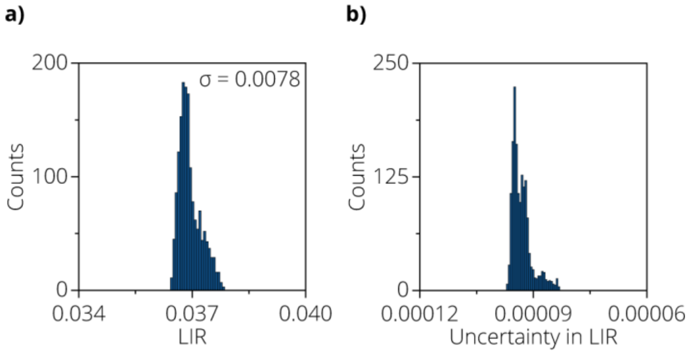

Figure S11. a) LIR values measured on the reduced sample at room temperature (294 K), without a gas flow. The measured area was 40x40 µm, to allow for more statistics. b) Calculated uncertainty in LIR due to random noise, using Eq. S7.

Theoretical background on ΔE and prefactor C

In our experiments Nd^{3+} acts as a "Boltzmann thermometer", because the populations of the emitting levels are in a thermal quasi-equilibrium.[10] The equation often quoted for the theoretical luminescence intensity ratio (LIR) from a single-ion Boltzmann thermometer is:[11]

$$\mathrm{LIR} = \frac{A_{20} g_2}{A_{10} g_1} \exp\left(-\frac{\Delta E_{21}}{k_\mathrm{B} T}\right) \tag{8}$$

where A_{i0} is the Einstein A coefficient for spontaneous emission from emitting level i (= 1,2) to the ground state, g_i is the degeneracy of level i, and ΔE_{21} is the energy difference between excited-state levels 1 and 2. Sometimes the energies $h\nu_i$ of emitted photons are included in the fraction, depending on whether the LIR is defined in terms of intensity (= energy per unit of time) or photon count rate (= number of photons per unit of time).

The factor $\frac{g_2}{g_1} \exp\left(-\frac{\Delta E_{21}}{k_\mathrm{B} T}\right)$ in Eq. S8 describes intrinsic properties of the thermometer, due to the energy-level structure of the emitting ions. The energy-level structure of lanthanide ions depends only weakly on any external influence and is determined by the local coordination of lanthanide ions in (nano)crystallites. Hence, if the luminescence thermometer is based on lanthanide emission, the factor $\frac{g_2}{g_1} \exp\left(-\frac{\Delta E_{21}}{k_\mathrm{B} T}\right)$ should be the same for ions in a specific crystalline material where the ΔE_{21} is fixed and will not vary in different sample environments, perhaps apart from minor differences in ΔE_{21} due to strain and/or external pressure.[12]

In contrast, the factor $\frac{A_{20}}{A_{10}}$ in Eq. S8 does *not* describe an intrinsic property of the thermometer. Einstein A coefficients are not constants but instead depend on the density of optical states as described by Fermi's golden rule.[13] This dependence gives rise to "local-field factors" in expressions for excited-state lifetime.[14] Also, scattering particles or reflective interfaces affect the density of optical states and, hence, the LIR.[15] Experiments record only a fraction of emitted light, namely the fraction that reaches the detector and is subsequently recorded.[16] This fraction may be affected by several factors, such as scattering, reflection, absorption, losses induced by the collection optics, and/or a limited efficiency of the detector. Any of these factors affect the LIR if and only if they affect the light emitted by states 1 and 2 differently. In practice, they often will.

Eq. S8 could be adapted to include the external factors on photon emission and recording. The influences of scattering and reflection could be described by the concept of a collected density of optical states, only considering emission into those photon states that reach the detector.[15] The influences of absorption, such as by biological tissue, could be described by a transmission factor.[16] Finally, the finite efficiency of the collection and detection system could be described by another efficiency factor. Taking all these effects into account, an adapted version of Eq. S8 would read:

$$\mathrm{LIR} = \frac{\rho_2 t_2 \eta_2 A_{20}^{(0)} g_2}{\rho_1 t_1 \eta_1 A_{10}^{(0)} g_1} \exp\left(-\frac{\Delta E_{21}}{k_\mathrm{B} T}\right) \quad (9)$$

Here, $A_{i0}^{(0)}$ is the Einstein A coefficient for spontaneous emission from level i to 0 in vacuum, ρ_i is the density of optical states at the emission wavelength of level i into the direction of the detector normalized to corresponding the density of optical states in vacuum, t_i is the transmission of the sample for light emitted by level i, and η_i is the collection and detection efficiency for light emitted by level i to 0.

We observe spatial variations in LIR in a reference measurement at a homogeneous constant temperature. We ascribe these to spatial variations in the factors described above. As our samples are both colored and scattering, we are not sure of the relative contributions from variations in t_2/t_1 (absorption) or from variations in ρ_2/ρ_1 (local-field effects). The relative contributions are not so relevant, because only the product of these contributions appears in Eq. S9. Our reference measurement at a homogeneous constant temperature calibrates the spatial dependence of the entire prefactor $\frac{\rho_2 T_2 \eta_2 A_{20}^{(0)} g_2}{\rho_1 T_1 \eta_1 A_{10}^{(0)} g_1}$, which is lumped together into parameter $C'(x, y)$ in Eq. 3 in the main text. This strategy of using a reference measurement at a known temperature is one of the solutions to avoid photonic artifacts proposed in Ref. [15].

Eq. S9 must be further adapted to account for background fluorescence from our samples. We arrive at a simple model equation if we assume that the background fluorescence has a fixed intensity relative to I_1—the intensity from the lower-energy thermometer level—and has a temperature-independent spectrum. This yields a recorded LIR of

$$\mathrm{LIR} = \frac{I_1 C \exp(-\Delta E_{21}/k_\mathrm{B} T) + b I_1 p_2}{I_1 + b I_1 p_1} \quad (10)$$

Here b is the ratio between background fluorescence intensity and I_1. p_1 and p_2 are the fractions of background fluorescence that overlap with I_1 and I_2, respectively. Eq. S10 can be rewritten to

$$\mathrm{LIR} = C' \exp\left(-\frac{\Delta E_{21}}{k_\mathrm{B}T}\right) + B \quad (11)$$

where the background-corrected prefactor C' is smaller than the background-free C of Eq. S9, because $b > 0$ for a positive background signal, and B is a constant that scales with the background intensity.

Different background characteristics than assumed in the derivation (e.g., a temperature-dependent intensity or spectrum) would yield a slightly different Eq. S10. Our derivation has the advantages of simplicity and some available interpretation of C' and B. Our model as well as other model equations yield approximately straight lines on the small temperature interval considered in our experiments, i.e., 25% change in absolute temperature between 540 and 680 K. Some previous studies used $\mathrm{LIR} = C'' \exp\left(-\frac{\Delta E'_{21}}{k_\mathrm{B}T}\right)$ as an approximate model, with an adapted energy gap between emitting levels $\Delta E'_{21}$. Similar to our model, this yields an approximate straight line of a small temperature interval, but variations of the value $\Delta E'_{21}$ have no good physical interpretation as lanthanide ions in the same host crystals experience the same local coordination.

Temperature maps at the other reactor set temperatures

The measurements were performed at three different reactor temperatures: 550, 595 and 640 K. The temperature maps from the measurement at 550 K are shown in Figure 3b/c, while the temperature maps for the other measurements are shown in Figure S12. At each temperature, the measurements were performed sequentially: (1) a measurement under Ar atmosphere, used for calibration (Figure 3a in the main text); (2) a measurement under CO_2/H_2/Ar flow, used to characterize local reaction-induced temperatures (Figure 3c); (3) a measurement under Ar atmosphere, used to confirm the uniform temperatures under inert conditions (Figure 3b).

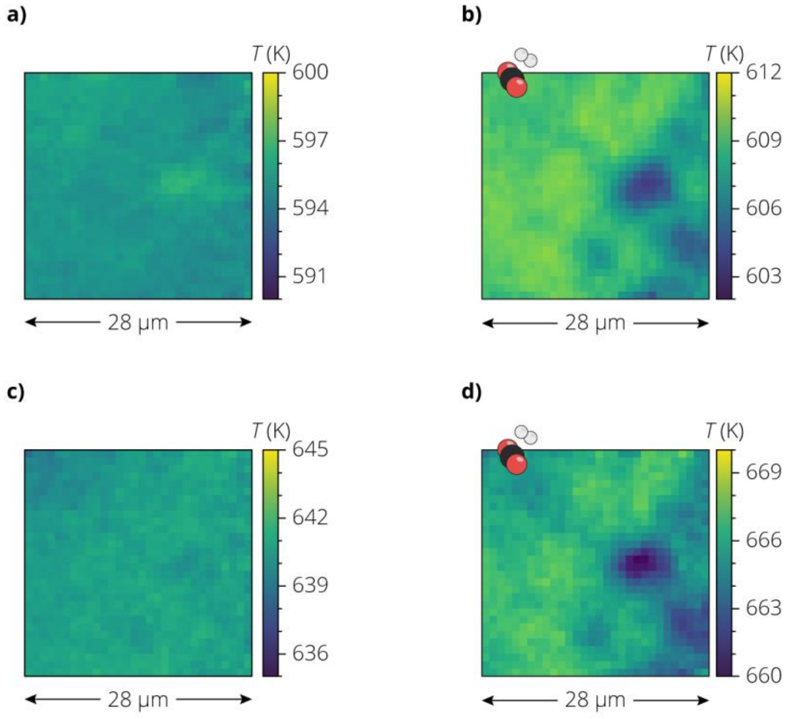

Figure S12. a) 2D map of the measured temperatures, calculated using the local calibration, in only Ar at a reactor temperature of 595 K. b) Same as in a), but after the introduction of CO_2 and H_2. c) 2D map of the measured temperatures, calculated using the local calibration, in only Ar at a reactor temperature of 640 K. d) Same as in c), but after the introduction of CO_2 and H_2.

Analysis of LIR maps: drift correction and uncertainty estimation

To perform the local calibration of $C'(x,y)$ and $B(x,y)$ on each pixel in the region of interest and then analyze the experiments under reaction conditions, we first correct for drift between the experiments. We use the pixel coordinates of the experiment at 550 K under inert conditions as a frame of reference. To determine the drift (X,Y) of a particular measurement with respect to the frame of reference, we maximize the correlation function:

$$\rho(X,Y) = \frac{\sum I_{\text{tot,ref}}(x,y) I_{\text{tot}}(x-X,y-Y)}{\sqrt{\sum I_{\text{tot,ref}}(x,y)^2 \sum I_{\text{tot}}(x-X,y-Y)^2}} \tag{12}$$

for (X,Y). Here $I_{\text{tot,ref}}$ is the total intensity (integrated over all wavelengths) in the reference experiment and I_{tot} is the linearly interpolated map of the total intensity in the measurement. The summation runs over all integer pixel values (x,y) of the reference experiments except for the pixels on the edge of the map. The pixels on the edge of the map are however used for the linear interpolation of I_{tot}. We find typical values of X and Y of 100–500 nm, i.e., 10–50% of a pixel (Figure S13). The drift (X,Y) is then used to define drift-corrected maps of I_1, I_2, and LIR:

$$I_1(x,y) = I_1'(x-X,y-Y) \tag{13}$$

$$I_2(x,y) = I_2'(x-X,y-Y) \tag{14}$$

$$\text{LIR}(x,y) = \frac{I_2(x,y)}{I_1(x,y)} \tag{15}$$

Here, I_1' and I_2' are the linearly interpolated maps of the integrated intensities of the two emission bands. Each calibration and actual measurement is separately drift-corrected with respect to the frame of reference. The drift-corrected calibration measurements of $\text{LIR}(x,y)$ are used in Figure 3a of the main text to calibrate the local values of $C'(x,y)$ and $B(x,y)$. The drift-corrected measurements at different reactor temperatures under different gas environments are used in Figure 3b–f to calculate local temperatures.

To estimate the uncertainties on the local temperatures, we first estimate the uncertainties on local-calibration fit parameters $C'(x,y)$ and $B(x,y)$. The sum of squared errors $\chi^2(C',B)$ between local-calibration model and the experimental LIR in calibration experiments (Figure 3a) is minimized for C' and B. The estimated errors on C' and B are calculated as:

$$\sigma_{C'} = \sqrt{2\chi^2(H^{-1})_{11}} \tag{16}$$

$$\sigma_B = \sqrt{2\chi^2(H^{-1})_{22}} \tag{17}$$

and the covariance:

$$\sigma_{C',B} = 2\chi^2(H^{-1})_{12} \tag{18}$$

where H is the Hessian matrix for $\chi^2(C', B)$. Both χ^2 and H are evaluated at the optimum values for C' and B. We find $\langle C' \rangle = 3.649$, $\langle B \rangle = 0.0287$, $\langle \sigma_{C'} \rangle = 0.014$, $\langle \sigma_B \rangle = 0.0013$, and $\langle \sigma_{C',B} \rangle = -0.00003$. The local temperature in a measurement is calculated from the measurement of LIR and from the locally calibrated C' and B,

$$T = \frac{-\Delta E}{k_{\mathrm{B}} \ln\left(\frac{\mathrm{LIR}' - B}{C'}\right)} \tag{19}$$

and the uncertainty in the temperature is estimated at:

$$\sigma_T = \sqrt{\left(\frac{\partial T}{\partial \mathrm{LIR}}\right)^2 \sigma_{\mathrm{LIR}}^2 + \left(\frac{\partial T}{\partial C'}\right)^2 \sigma_{C'}^2 + \left(\frac{\partial T}{\partial B}\right)^2 \sigma_B^2 + 2\frac{\partial T}{\partial C'}\frac{\partial T}{\partial B}\sigma_{C',B}} \tag{20}$$

For the experiments under reaction atmosphere, we find $T = 556.3 \pm 1.4$ K and $\sigma_T = 0.35 \pm 0.15$ (mean $\pm$ standard deviation over 28×28 pixels, excluding the pixels on the edge) at a reactor temperature of 550 K; $T = 608.6 \pm 1.3$ K and $\sigma_T = 0.32 \pm 0.10$ at a reactor temperature of 595 K; and $T = 665.7 \pm 1.4$ K and $\sigma_T = 0.45 \pm 0.21$ at a reactor temperature of 640 K.

For the experiments under inert atmosphere (measured after the reaction), we find $T = 550.1 \pm 0.3$ K and $\sigma_T = 0.37 \pm 0.17$ at a reactor temperature of 550 K; $T = 595.3 \pm 0.4$ K and $\sigma_T = 0.32 \pm 0.09$ at a reactor temperature of 595 K; and $T = 640.6 \pm 0.4$ K and $\sigma_T = 0.40 \pm 0.15$ at a reactor temperature of 640 K.

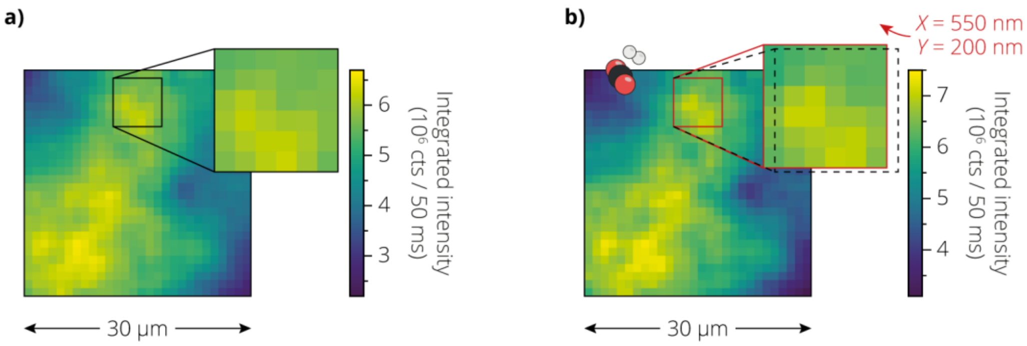

Figure S13. a) 2D map of the total Nd^{3+} emission intensity I_{tot}, integrated over all wavelengths between 795 and 980 nm, of the frame-of-reference experiment (reactor temperature 550 K; inert atmosphere). b) Same as in a), but in the $CO_2/H_2/Ar$ gas atmosphere. The map in b) must be shifted by a small amount for the highest correlation with the map in a), as indicated in the inset.

Temperature variations due to oscillations in gas feed

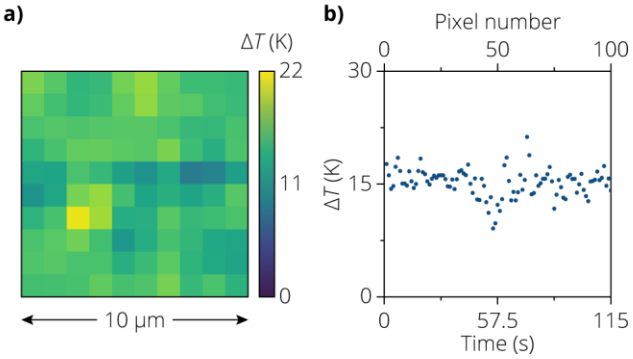

Figure S14. a) 2D map of the measured luminescence intensity ratios (LIR) under $CO_2/H_2/Ar$ atmosphere on a 10×10 μm area at a reactor temperature of 640 K. Integration time per pixel was 1 s. b) Measured temperature increase (ΔT) as a function of pixel number and measurement time in a confocal scan. The pixel numbers were counted from top left to bottom right, as measured.

Temperature deviation of Harrick HVC cell

The Harrick cell consists of an internal thermocouple at position **X** in Figure S15a. This thermocouple is placed closer to the heating rod than the sample and it therefore overestimates the temperature of the sample. We placed an extra thermocouple through the gas outlet, ending up at position **Y** in Figure S15a. We measured the temperatures at both position in a temperature range between room temperature and 873 K. The deviation from the blue dotted line in Figure S14b shows that the sample temperature is deviating from the readout temperature at position **X**. We used the temperature values at position **Y** for our calculations.

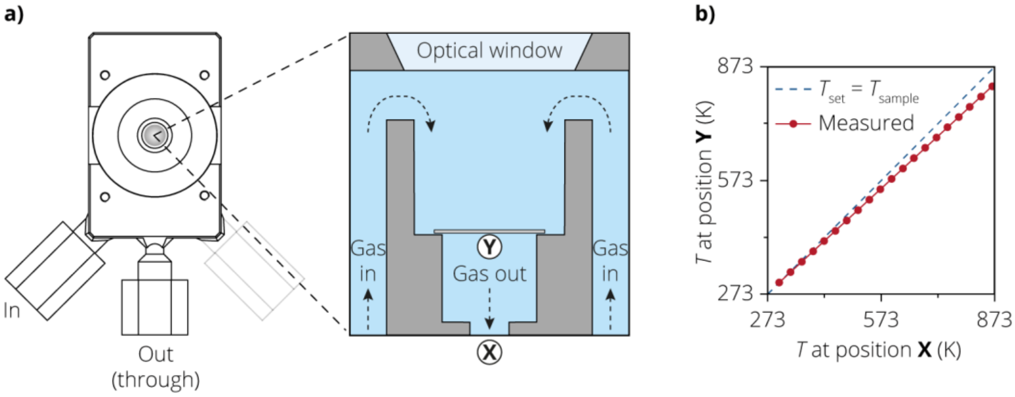

Figure S15. a) Drawing of the Harrick HVC cell, with a zoom in on the reaction chamber. The self-supporting wafer is placed at position **Y**, while the heating element and the internal thermocouple are placed at position **X**. b) Measured temperatures with an external thermocouple placed at position **Y**, compared to the readout values of the internal thermocouple at position **X**.

Laser heating due to sample blackening

The blackening of the sample could lead to heating of the sample due to laser absorption. We checked whether the laser power used for the thermometry experiments (1% relative to the maximum power) was sufficient to facilitate this heating. Figure S16 shows the results of this measurement, where the LIR is constant below 10%, indicating that the 1% laser power used in the experiments is safe to prevent laser heating.

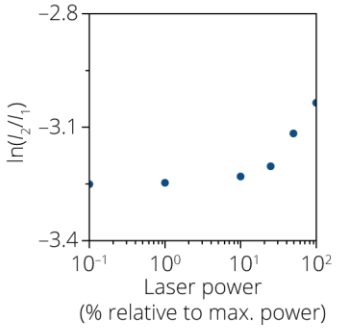

Figure S16. The effect of the laser power of the Horiba Raman spectrometer on the LIR, measured at room temperature on the blackened sample. The laser power of 1% (relative to the maximum power) used in the thermometry experiments during CO_2 methanation does not influence the measured LIR, while laser powers > 10% affect the LIR in a way consistent with illumination-induced heating.

References

1. Vetrone, F., Naccache, R., Zamarrón, A., de la Fuente, A. J., Sanz-Rodríguez, F., Maestro, L. M., Rodriguez, E. M., Jaque, D., Solé, J. G., Capobianco, J. A. Temperature sensing using fluorescent nanothermometers. *ACS Nano* **4**, 3254–3258 (2010).
2. Rabouw, F. T., Den Hartog, S. A., Senden, T. & Meijerink, A. Photonic effects on the Förster resonance energy transfer efficiency. *Nat. Commun.* **5**, 3610 (2014).
3. van Swieten, T. P., Yu, D., Yu, T., Vonk, S. J. W., Suta, M., Zhang, Q., Meijerink, A., Rabouw, F. T. Ho^{3+}-Based Luminescent Thermometer for Sensitive Sensing over a Wide Temperature Range. *Adv. Opt. Mater.* **9**, 2001518 (2020).
4. Maciejewska, K., Bednarkiewicz, A. & Marciniak, L. The influence of the Er^{3+} dopant concentration in $LaPO_4$:Nd^{3+}, Er^{3+} on thermometric properties of ratiometric and kinetic-based luminescent thermometers operating in NIR II and NIR III optical windows. *Phys. B Condens. Matter* **620**, 413247 (2021).
5. Dramićanin, M. D. *Luminescence Thermometry - Methods, Materials and Applications*. (Elsevier, Amsterdam, 2018).
6. van Swieten, T. P., Meijerink, A. & Rabouw, F. T. Impact of Noise and Background on Measurement Uncertainties in Luminescence Thermometry. *ACS Photonics* **9**, 1366–1374 (2022).
7. Carnall, W. T., Crosswhite, H. & Crosswhite, H. M. *Energy level Structure and Transition probabilities in the Spectra of Trivalent Lanthanides in* LaF_3. (Argonne National Laboratory, Lemont, 1978).
8. Vetrone, F., Boyer, J. C., Capobianco, J. A., Speghini, A. & Bettinelli, M. Effect of Yb^{3+} codoping on the upconversion emission in nanocrystalline Y_2O_3:Er^{3+}. *J. Phys. Chem. B* **107**, 1107–1112 (2003).
9. Ubaldini, A. & Carnasciali, M. M. Raman characterisation of powder of cubic RE_2O_3 (RE = Nd, Gd, Dy, Tm, and Lu), Sc_2O_3 and Y_2O_3. *J. Alloys Compd.* **454**, 374–378 (2008).
10. Suta, M., Antic, Z., Djordevic, V., Kuzman, S., Dramicanin, M. D., Meijerink, A. Making Nd^{3+} a Sensitive Luminescent Thermometer for Physiological Temperatures — An Account of Pitfalls in Boltzmann Thermometry. *Nanomaterials* **10**, 543 (2020).
11. Suta, M. & Meijerink, A. A Theoretical Framework for Ratiometric Single Ion Luminescent Thermometers—Thermodynamic and Kinetic Guidelines for Optimized Performance. *Adv. Theory Simulations* **3**, 2000176 (2020).
12. Balabhadra, S., Debasu, M. L., Brites, C. D. S., Ferreira, R. A. S. & Carlos, L. D.

Upconverting Nanoparticles Working As Primary Thermometers in Different Media. *J. Phys. Chem. C* **121**, 13962–13968 (2017).

13. Novotny, L. & Hecht, B. *Principles of Nano-Optics*. (Cambridge University Press, Cambridge, 2012).
14. Henderson, B. & Imbusch, G. F. *Optical Spectroscopy of Inorganic Solids*. (Taylor & Francis, London, 1989).
15. Vonk, S. J. W., van Swieten, T. P., Cocina, A. & Rabouw, F. T. Photonic Artifacts in Ratiometric Luminescence Nanothermometry. *Nano Lett.* **23**, 6560–6566 (2023).
16. Shen, Y., Lifante, J., Fernández, N., Jaque, D. & Ximendes, E. In Vivo Spectral Distortions of Infrared Luminescent Nanothermometers Compromise Their Reliability. *ACS Nano* **14**, 4122–4133 (2020).
